# A mobile observatory powered by sun and wind for near real time measurements of atmospheric, glacial, terrestrial, limnic and coastal oceanic conditions in remote off-grid areas

**DOI:** 10.1016/j.ohx.2022.e00331

**Published:** 2022-06-20

**Authors:** Søren Rysgaard, Kim Bjerge, Wieter Boone, Egon Frandsen, Michael Graversen, Toke Thomas Høye, Bjarne Jensen, Geoffrey Johnen, Marcin Antoni Jackowicz-Korczynski, Jeffrey Taylor Kerby, Simon Kortegaard, Mikhail Mastepanov, Claus Melvad, Peter Schmidt Mikkelsen, Keld Mortensen, Carsten Nørgaard, Ebbe Poulsen, Tenna Riis, Lotte Sørensen, Torben Røjle Christensen

**Affiliations:** a Arctic Research Centre, Aarhus University, Aarhus C, Denmark; b VLIZ - Flanders Marine Institute (VLIZ), Oostende, Flamish Region, Belgium; c MG Solar, Frederiksborgvej 399, Roskilde, Denmark; d Aarhus Institute of Advanced Studies, Aarhus University, Aarhus C, Denmark; e Mopa Patrol Både, Møllevej 21, Vilsund, Denmark; f Department of Biology, Aarhus University, Denmark; g Department of Mechanical and Production Engineering, Aarhus University, Denmark; h Department of Environmental Science, Aarhus University, Denmark; i Department of Ecoscience, Aarhus University, Denmark; j Department of Physical Geography and Ecosystem Science, Lund University, Sweden; k Centre for Earth Observation Science, University of Manitoba, Canada; l Greenland Institute of Natural Resources, Greenland; m Oulanka Research Station, Oulu University, Finland; n Department of Electrical and Computer Engineering, Aarhus University, Denmark

**Keywords:** Remote, Arctic, Environment, Observatory, Autonomous, Near-real time

## Abstract

Climate change is rapidly altering the Arctic environment. Although long-term environmental observations have been made at a few locations in the Arctic, the incomplete coverage from ground stations is a main limitation to observations in these remote areas. Here we present a wind and sun powered multi-purpose mobile observatory (ARC-MO) that enables near real time measurements of air, ice, land, rivers, and marine parameters in remote off-grid areas. Two test units were constructed and placed in Northeast Greenland where they have collected data from cabled and wireless instruments deployed in the environment since late summer 2021. The two units can communicate locally via WiFi (units placed 25 km apart) and transmit near-real time data globally over satellite. Data are streamed live and accessible from (https://gios.org). The cost of one mobile observatory unit is *c.* 304.000€. These test units demonstrate the possibility for integrative and automated environmental data collection in remote coastal areas and could serve as models for a proposed global observatory system.

## Specifications table

**Table:** 

Hardware name	ARC-MO (Arctic Research Centre – Mobile Observatory)
Subject area	Environmental, planetary and agricultural sciences
Hardware type	Field measurements and sensors
Closest commercial analog	No commercial analog is available*.*
Open source license	CC BY 4.0
Cost of hardware	*304.000€*
Source file repository	https://doi.org/10.17632/5gfmy98kcj.2

## Hardware in context

Arctic surface temperatures have increased at three times the global average [1]. Its cascading effects on the environment remain uncertain, with widespread implications for plants, animals, and people in sparsely populated or remote areas and beyond. It has become increasingly clear that large differences in climatic conditions can emerge across local scales and that it is very difficult to predict seasonal and interannual variability in a range of parameters that span the domains of air, ice, land, rivers, and coastal oceans. New autonomous data collection approaches are needed for remote and off-grid regions to better upscale local findings, validate models, and to ground truth and calibrate satellite datasets [[2], [3]]. A growing interest in upscaling local terrestrial observations to the pan-Arctic has resulted in an international collaboration between Arctic research stations surrounding the Arctic Ocean [4]. In the marine Arctic, few large offshore subsea observatories exist that focus on long-term observations, such as the LTER Observatory Hausgarten [5] in the Fram Strait and the Global Irminger Sea array [6]. For scaling up measurements, ice-tethered profilers are being deployed in sea ice that facilitate for more comprehensive oceanic sampling by moving up and down in the water column [7]. Recently, a few smaller coastal undersea observatories have been deployed in communities of the Canadian Arctic. A typical community observatory installation includes an underwater instrument platform located on the ocean floor and linked by cable to a nearby wharf connection [8]. Presently, there is need for development of mobile autonomous off-grid observatories that can monitor the atmosphere-land–ocean transition. In addition, theses observatories should be able to function year-round in coastal areas such as around the Greenland ice sheet where conditions make access and monitoring challenging due to harsh Arctic weather conditions and the presence of wildlife, sea ice, and icebergs. The Arctic Research Centre Mobile Observatory (ARC-MO) addresses these challenges and is a result of an interdisciplinary collaboration between atmospheric, terrestrial, limnic, ocean and technical scientists in the newly established Greenland Integrated Observing System infrastructure project (https://gios.org).

## Hardware description

Our project requirements for the mobile observatory and the results are outlined in Table 1.

**Table 1:** Desired product requirements and their status in the final technical specifications.

Product requirements		Achieved?
**1. Usage**		
Deployment	From helicopter and small vessel	yes
Handling	2–3 people	yes
Operation	1–2 years	not fully tested
**2. Transport**		
Shipping	6-foot insulated aluminium container	yes
**3. Environment**		
Air temperature	−35 to 40 °C	yes
Water temperature	−2 to 20 °C	yes
**4. Design**		
Dimensions	6 foot containers covered by 4 solar panels and windmill	yes
Weight	< 1200 kg per unit	yes
**5. Power production**		
Atmosphere and land unit	up to 2000 Wh per day	yes
Atmosphere and marine unit	up to 1200 Wh per day	yes
Battery bank	> 30 kWh. Capacity to sustain the entire system for 12–18 days without charging.	yes
**6. Example of data collection setup**		
Atmosphere	Air and skin temperature Atmospheric pressure PAR Net and global radiation Relative humidity Wind speed and direction Momentum fluxes Sensible and latent heat fluxes CO_2_ fluxes CH_4_ fluxes	yes
Land	Air temperature Relative humidity Air pressure Net and global radiation PAR Snow depth Precipitation Wind speed and direction Sensible and latent heat flux Surface temperature Soil temperatures CO_2_ fluxes (EC and chamber) CH_4_ fluxes (chamber) Phenocams (timing of snowmelt, plant phenology and insect activity)	yes
Limnic	Water temperature Water level pressure Conductivity Water turbidity pH O2 Light intensity Photos (stream reach and in-stream)	Fully implemented in 2022
Ocean	Ice keel (sea ice thickness) Currents Temperature Salinity Tide PAR Fluorescence (chl)	yes
**7. Data transmission**		
Data storage	All data collected are stored on local server	yes
Cable	Data from the marine sensors are transmitted by inductive link and 4 mm steel cable to the server in the container with a distance up to 1–2 km.	yes
WiFi	Data from the field (land and limnic) is transmitted via WiFi to the server in the container	yes
	WiFi connection between units (ca 25 km)	yes
Satellite telemetry	Average data (hourly) is transmitted (by FTP) once a day	yes

**Fig. 1:**
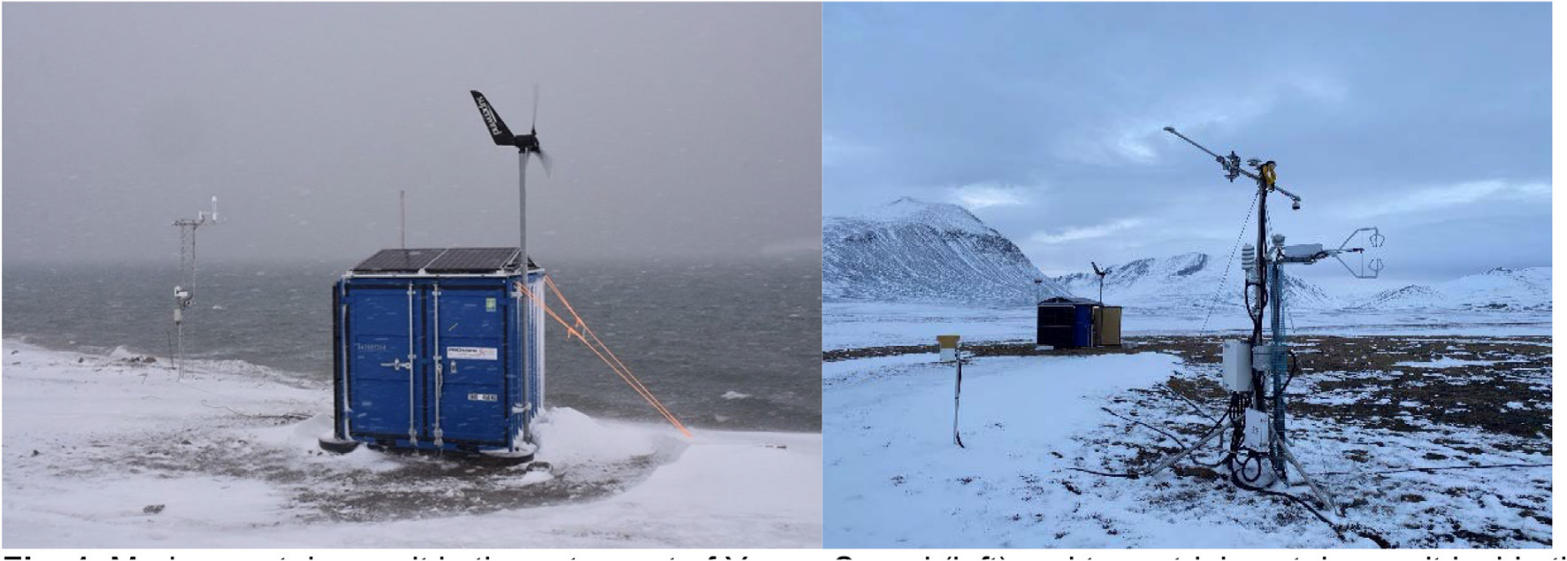
Marine container unit in the outer part of Young Sound (left) and terrestrial container unit inside the fjord near the Zackenberg Research Station (right) in NE Greenland (74°N). The marine unit collects data from the meteorological mast in the background and from the fjord ca 1 km off the coast. The terrestrial container collects data from the meteorological mast in the foreground and from various installations in the landscape.

**Fig. 2:**
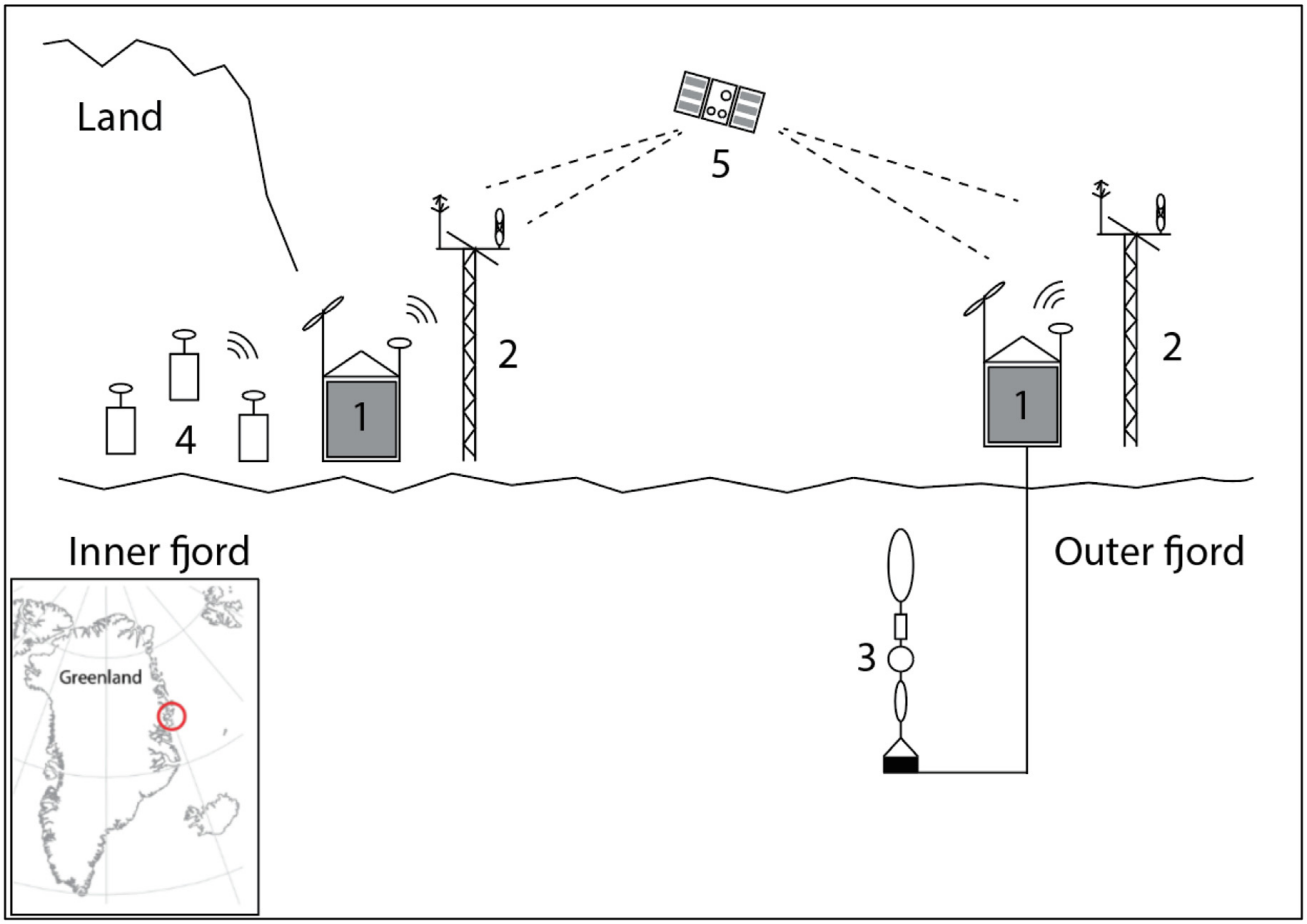
Test site in NE Greenland (Inserted lower left). Red circle is enclosing the Young Sound-Tyrolerfjord system and the Zackenberg/Daneborg research stations. Operational concept of ARC-MO consisting of (1) container units with solar panels, windmill and transmitting systems (2) meteorology masts (3) cabled subsea instruments (4) various instruments on land and lakes e.g. cameras, thermistors, gas flux systems, runoff sensors and (5) satellite communication. The ARC-MO units are also able to communicate with each other through WiFi over 25 km. (For interpretation of the references to colour in this figure legend, the reader is referred to the web version of this article.)

Development resulted in the ARC-MO shown in Fig. 1 and Fig. 2. The two systems were deployed at off-grid conditions in the Young Sound-Tyrolerfjord system located at *c.* 74°N; 20°W in Northeast Greenland. This area is the site of the Zackenberg research station part of the Greenland Ecological Research Operations (https://www.g-e-m.dk; [[9], [10]] that cover long-term data collection of a high-Arctic environment. Our idea is to extend long-term observations of key parameters beyond the station area to several locations around Greenland.

## Container

**Fig. 3:**
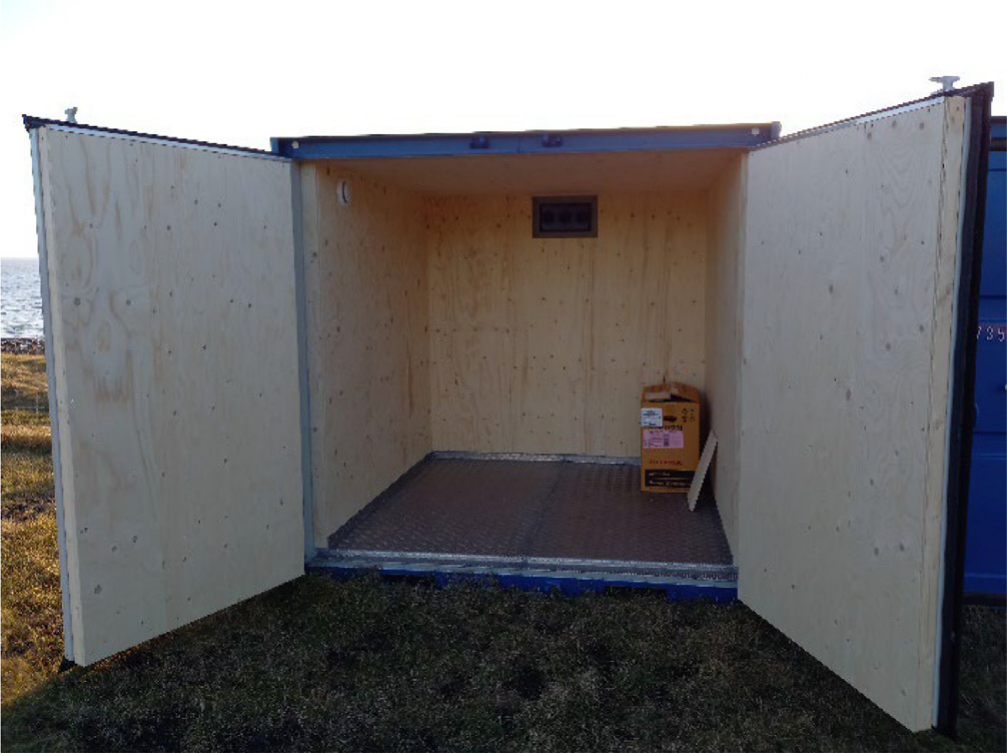
The inner part of an empty container (left). Inlets on the rear of the container (right).

Each container is a modified 6 ft shipping container (Fig. 3). The dimensions are as follows: outer dimensions L 200 cm, W 200 cm, H 202 cm. Inner dimensions: L:165 cm, W 170 cm, H ca. 165 cm. The front of the container consists of 2 doors with the door to the right used as “main” door. The containers are insulated with glass wool, 50–70 mm on the sides and doors, and 100 mm on the ceiling, with 15 mm plywood on the ceiling and walls. The floor is insulated with 25 mm styrofoam bats and covered by a 3/5 non-slip aluminium floorboard. A 27 × 36 cm hole was added to the rear center top of the container. This hole is fitted with a plate with three “inlet” ø50 mm pipe vents. All pipe vents are facing downwards and filled with insulation material to avoid fine snow etc. coming into the container.

## Power production

**Fig. 4:**
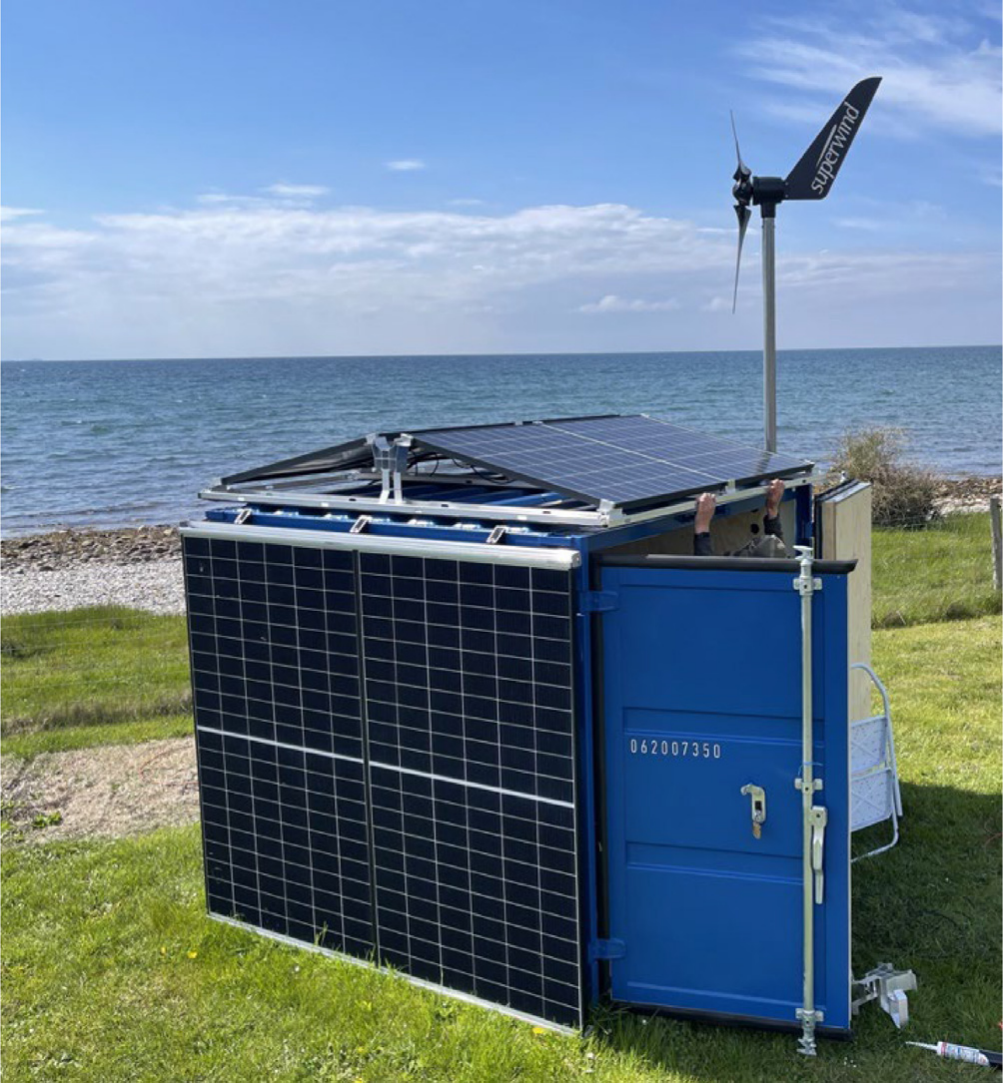
Solar panels and a wind turbine mounted on a container (at test site in Denmark).

Energy production was sourced from solar and wind, as both resources are readily available under arctic conditions at various times of the year and recent technological advances have made these power sources well suited for supplying off grid systems (Fig. 4). During the summer, solar energy is abundant due to the midnight sun providing a steady power source. We found that during the summer, the combination of solar and wind turbines presented no issues with keeping the system charged, whereas during the winter period, when the power output from the solar panels falls to zero during the polar night, consistent power supply is more challenging due to the sole dependency on wind.

## Solar power

There are two solar panels (facing south) and 2 solar panels on the roof for each container. The solar modules are a class of modules known as *dual-string modules*, with two parallel strings of cells, connected in the middle of the module. All solar panels are type REC Alpha Series, model 380 WP Power. The size of each panel is: 172.1 × 101.6 × 0.30 cm, weight: 19.5 kg. This type of module was chosen for both its high power and for its capability to resist shadow effects. It is expected that snow will pile up against the containers and thus shadow the vertically mounted modules. For dual-string modules the vertically mounted modules perform at 50 % capacity when covered by snow up to 85–90 cm. deep, whereas a more commonly used layout of solar modules would see a power cut off entirely with snow coverage of < 20 cm. The solar modules are connected in serial per pair with one series being the vertically mounted modules and the other the two modules on the roof. The vertical solar modules are mounted with an aluminium insert system and the roof modules are mounted in an east/west-system.

## Wind power

The selected wind turbines, Superwind 353 from Superwind GmbH, mounted on a ø60 mm pole on the corner of the container, each have a power output of up to 350 W and dual bearings to resist the harsh and changing conditions they will be exposed to. The turbine has 3 carbon/glass fibre reinforced plastic blades and covers an area of 1.17 m^2^ (diameter of 122 cm). The minimum space required for free turning (360°) is 126.3 cm. The cut in wind speed is 3.5 m/s with no upper limit. The nominal voltage is 24 VDC, and nominal power is 350 W at 12.5 m/s. The turbines have a “dump off” of power that will serve as an occasional heat source in the container when batteries are fully charged as well as protection from excess wind conditions. This first setup has one wind turbine per container unit, but we will increase this to two in the next version to ensure sufficient power generation in over the dark winter period.

## Power storage

**Fig. 5:**
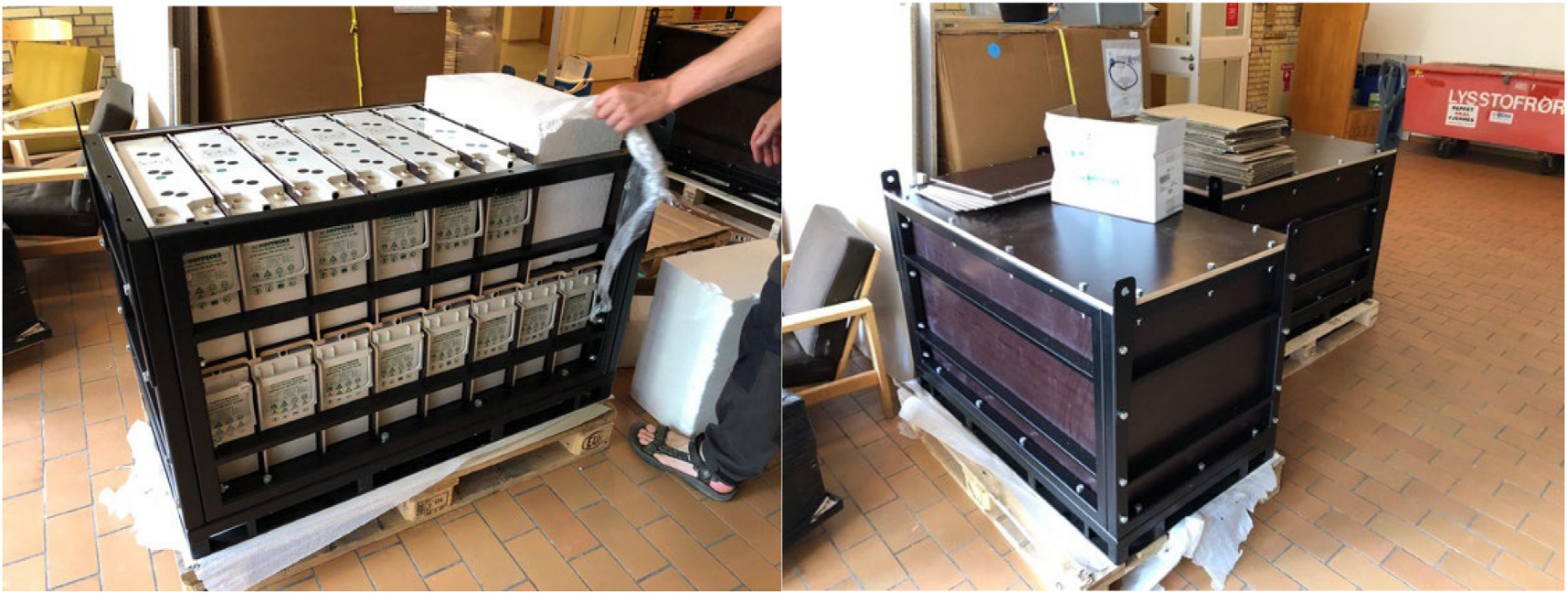
Battery cage open (left) and closed for transport with helicopter (right).

The power storage consists of 14 lead-acid batteries with a rated capacity of 180 Ah/12 V (Fig. 5), resulting in a total capacity of 30 kWh. The size of each battery is 55.1 × 12.5 × 30.5 cm and weigh 64.4 kg. The batteries supply the power for the monitoring equipment and are equipped with a shunt resistor to track the state of charge with this output entering a Victron Smart Battery Protect System that shuts down the system when it falls to a state of charge of approximately 40 % to not completely discharge batteries and thereby preserve longevity. The lead batteries are chosen for their well-established durability in diverse conditions (temperature range, discharge rate) and though more suitable technologies exist, lead batteries are a solid compromise between performance and cost. Specifically, Li-ion batteries will see a drop off in charge acceptance at sub-zero temperatures reaching a cut off at the temperatures this system commonly experiences. For stowing, a custom-made rack is designed that also serve as transport encasing while hoisting the batteries in with helicopter to desolate locations. Batteries are series connected in pairs to a system voltage of 24 V with the same output feeding into all power converters.

## Transportation and setup of container units

The containers were transported to Greenland by a Danish Navy vessel and slung into place by helicopter. Onsite installation is done by a technical team.

## Energy consumption (monthly).

**Fig. 6:**
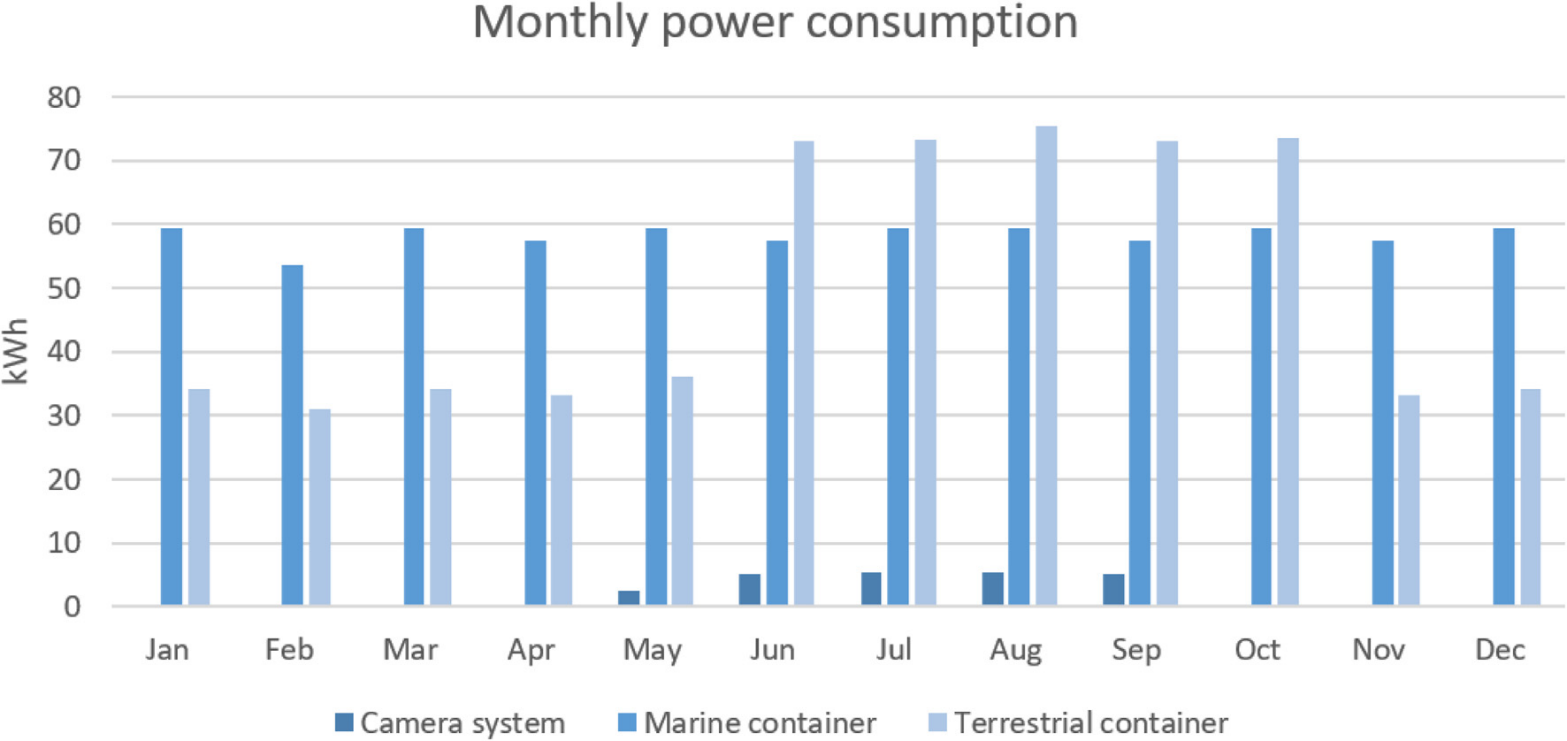
Monthly power consumption (kWh) in two different container systems.

An example of the monthly power consumption in two different configured container systems is shown in Fig. 6. Details are found in supplementary (Power_consumption.xlsx). Power intensive equipment is only powered on when needed, e.g. the satellite system, routers and antennae. Other sensors, like the camera system (having their own power system) and chambers are turned off in the wintertime to reduce power consumption. The system is flexible and can easily be configured to adapt to different needs and environments.

## Wiring system

**Fig. 7:**
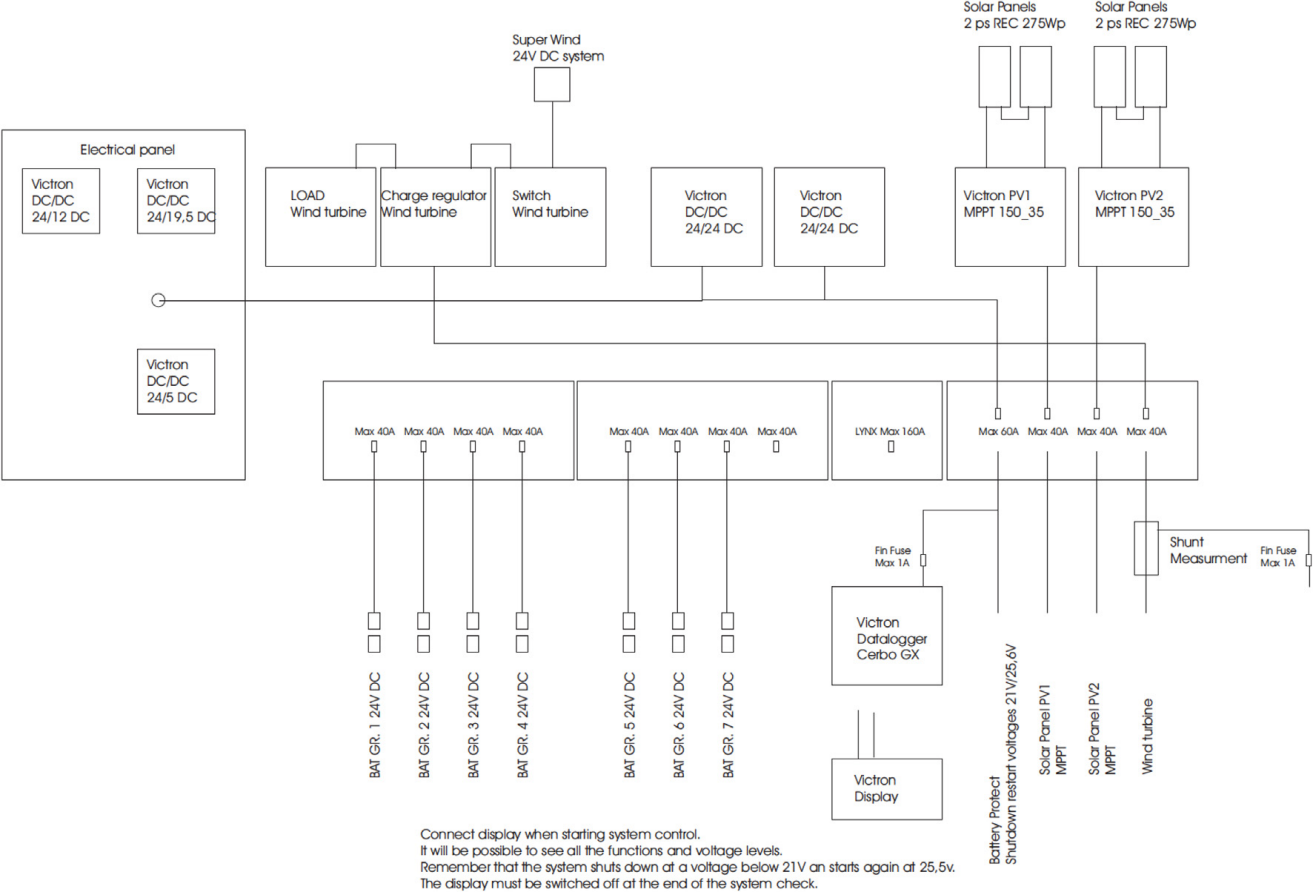
Wiring diagram of the power supply system.

The wiring diagram of the ARC-MO unit is presented in Fig. 7. The supply system can be remotely monitored through the Cerbo GX datalogger that also has wireless data transfer capabilities.

## Data collection

Data collection is divided into three compartments:

• Atmosphere
• Land (including streams)
• Ocean

## Atmosphere

**Fig. 8:**
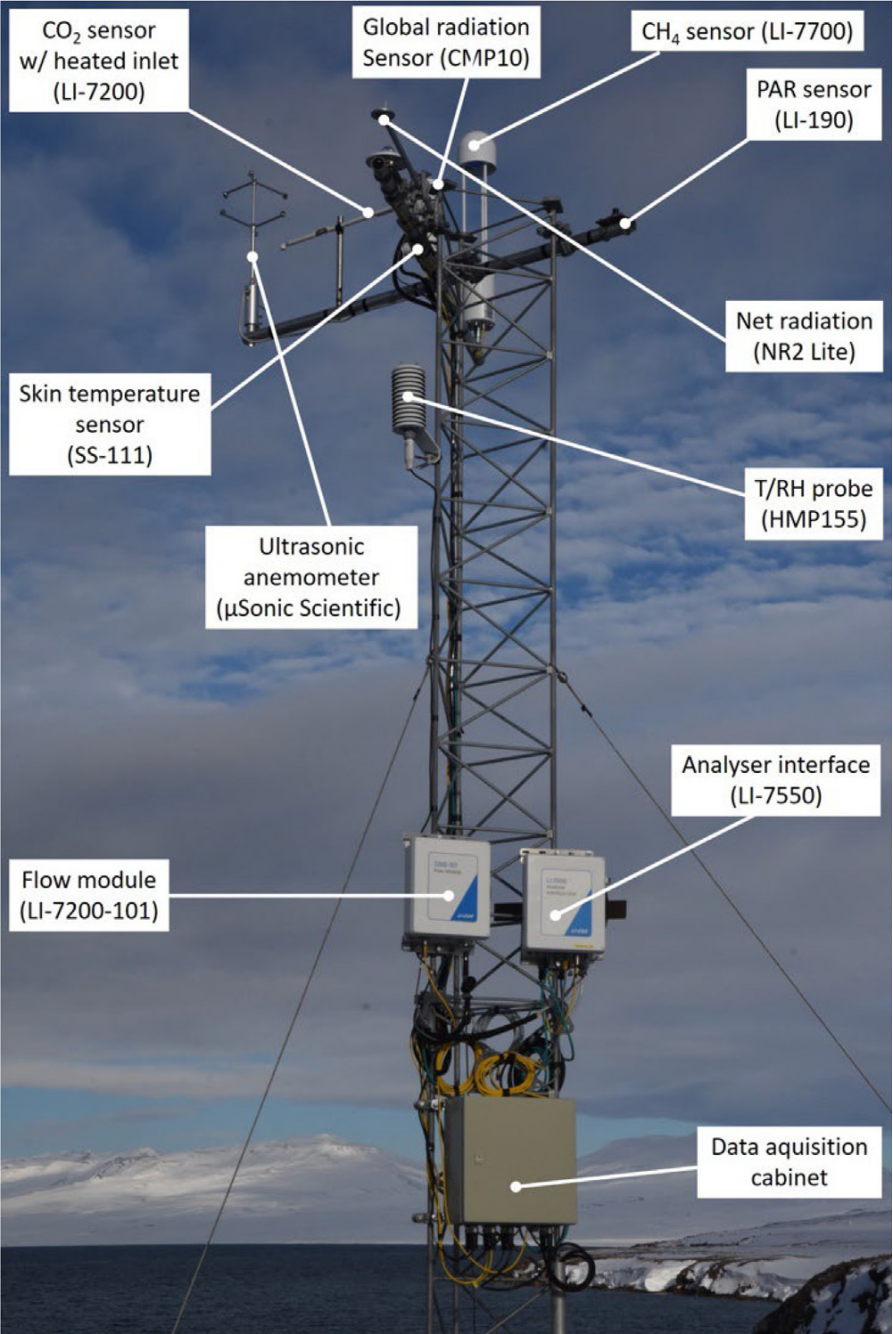
Atmospheric instruments in the 6 m mast.

Instruments for measurements of climatology, meteorology and surface fluxes are installed on a 6-meter mast approximately 20 m from the container (Fig. 8). The mast consists of two 3-meter sections, Carl C model B450. It is an equilateral triangle, where each side of the mast section are 45 cm wide. The mast is installed on a triangular metal plate and supported by three 6 mm guywires and stakes dug into the ground. The mast is transported in 3 m sections and assembled on site. All equipment, including the two smaller container units, were fitted into a 20 ft container and shipped to Daneborg in Northeast Greenland. We used a helicopter to transport to the exact deployment locations in Daneborg and in Zackenberg (25 km apart).

### Ambient relative humidity and air temperature

The ambient relative humidity and air temperature is measured with a HMP155 Humidity and Temperature probe manufactured by Vaisala Oyj, Finland. The scaling for the relative humidity is 0 to 100 %, and for the temperature −80 to + 60 °C. Both channels have an output of 0 to 1 V. The probe is mounted directly on the vertical leg on the mast in a Vaisala DTR503 radiation shield. The measuring hight is 5.2 m above ground.

### Skin temperature

The skin temperature is measured with an Infrared Radiometer, model SI-100-SS manufactured by Apogee Instruments, Utah, USA. The sensor has an operation temperature of −55 to + 80 °C, 0 to 100 % RH. Analog output is 0 to 2500 mV. The sensor is mounted on top of the mast (6 m above ground) in an angle to measure the skin temperature of the fjord.

### PAR (Photosynthetically active Radiation)

PAR is measured with Quantum Sensor, model LI-190/R from Li-Cor Bioscience, Nebraska, USA. Operating temperature for this sensor is −40 to + 65 °C. Output is 7 µA/1000 µmol s^−1^ m^−2^. The PAR sensor is mounted on the mast at 6 m height above ground on a ø50 mm aluminium-boom pointing towards east.

### Net radiation

Net radiation is measured with a Net Radiometer model NR Lite 2 manufactured by Kipp & Zonen B.V., The Netherlands. The spectral range is 0.2 to 100 µm, and the operating temperature is −40 to + 80 °C. The output is a low-level voltage output ranging from 0 to 15 mV. The sensor is mounted as an extension of a ø50 mm aluminium boom approximately 1.3 m away from the mast and at 6 m height above ground.

### Global radiation

The global radiation is measured with a CMP 10 pyranometer manufactured by Kipp & Zonen B.V., The Netherlands. The spectral range is 285 to 2800 µm, and the operating temperature is −40 to + 80 °C. Signal output is 0 to 20 mV. The global radiation sensor is installed at the mast on a ø50 mm aluminium-boom at 6 m height.

### Data logging of meteorological parameters

All radiation instruments, temperature and humidity sensors are connected to a Campbell datalogger, model CR1000X for continuous data collection. The datalogger is connected to a server in the marine container with an approximately 28 m long cable.

## Wind speed and direction, momentum fluxes and sensible heat fluxes

The 3-D wind components and temperature are measured at high frequency (10 Hz) with an ultrasonic anemometer Model uSonic-3 Scientific manufactured by METEK Meteorologische Messtechnik GmbH, Germany. The sensor head and the electronics are separated, and the anemometer is equipped with sensor head heating which can be turned on/off. It has analogue as well as digital data output (0 to 5 VDC & RS422), and in this setup the analogue output is used for data transmission. The parameter settings are: u: −60 to + 60 m s^−1^, v: −60 to + 60 m s^−1^, w: −5 to + 5 m s^−1^, T: −50 to + 50 °C. The ultrasonic anemometer is mounted at the end of a ø50 mm aluminium-boom in the mast at 6 m height, and the boom pointing towards the fjord (towards west).

### CO_2_ and latent heat fluxes

A LI-7200/RS Enclosed CO_2_/H_2_O gas analyzer is used in connection with a LI-7200–101 Flow Module and a heated inlet to measure fluxes of H_2_O and CO_2_. The system is manufactured by Li-Cor Bioscience, Nebraska, USA. The calibration range for CO_2_ is 0 to 3000 µmol mol^−1^, and for H_2_O 0 to 60 µmol mol^−1^. The operation temperature is −25 to + 50 °C. The output is 0 to 5 V and the power consumption is max. 37 W during warm-up, and 8 to 18 W steady state. The CO_2_/H_2_O sensor is installed in the mast at 6 m height. The sensor inlet is pointing towards the fjord (towards west) along the 50 mm aluminium-boom containing the ultrasonic anemometer. The LI-7200–101 Flow Module is mounted at the mast in 2.5 m height.

### CH_4_ fluxes

A LI-7700 Open Path CH_4_ gas analyzer, manufactured by Li-Cor Bioscience, Nebraska, USA, is used to measure fluxes of CH_4_. The calibration range for CH_4_ is 0 to 40 ppm @ +25 °C, and 0 t0 25 ppm @ −25 °C. The operation temperature is −25 to + 50 °C. The output is 0 t0 5 V. Power consumption is 8 W nominally. The CH_4_ sensor is installed on a ø50 mm aluminium-boom pointing towards south in the mast at 6 m height.

### Data collection of heat, CO_2_ and CH_4_ fluxes

The uSonic-3 Scientific, the LI-7200/RS and the LI-7700 are connected to a LI-7550 Analyzer Interface Unit manufactured by Li-Cor Bioscience, Nebraska, USA for setup control and data collection. The LI-7550 has a 4 GB removable USB storage, an ethernet connection, RS-232 (57.600 baud, 20 records s^−1^) and 6 Digital-to-Analog converters (0–5 V). The operation temperature is −25 to + 50 °C. Power consumption is 10 W nominally. The LI-7550 AIU is mounted at the mast in 2.5 m height.

## Land

**Fig. 9:**
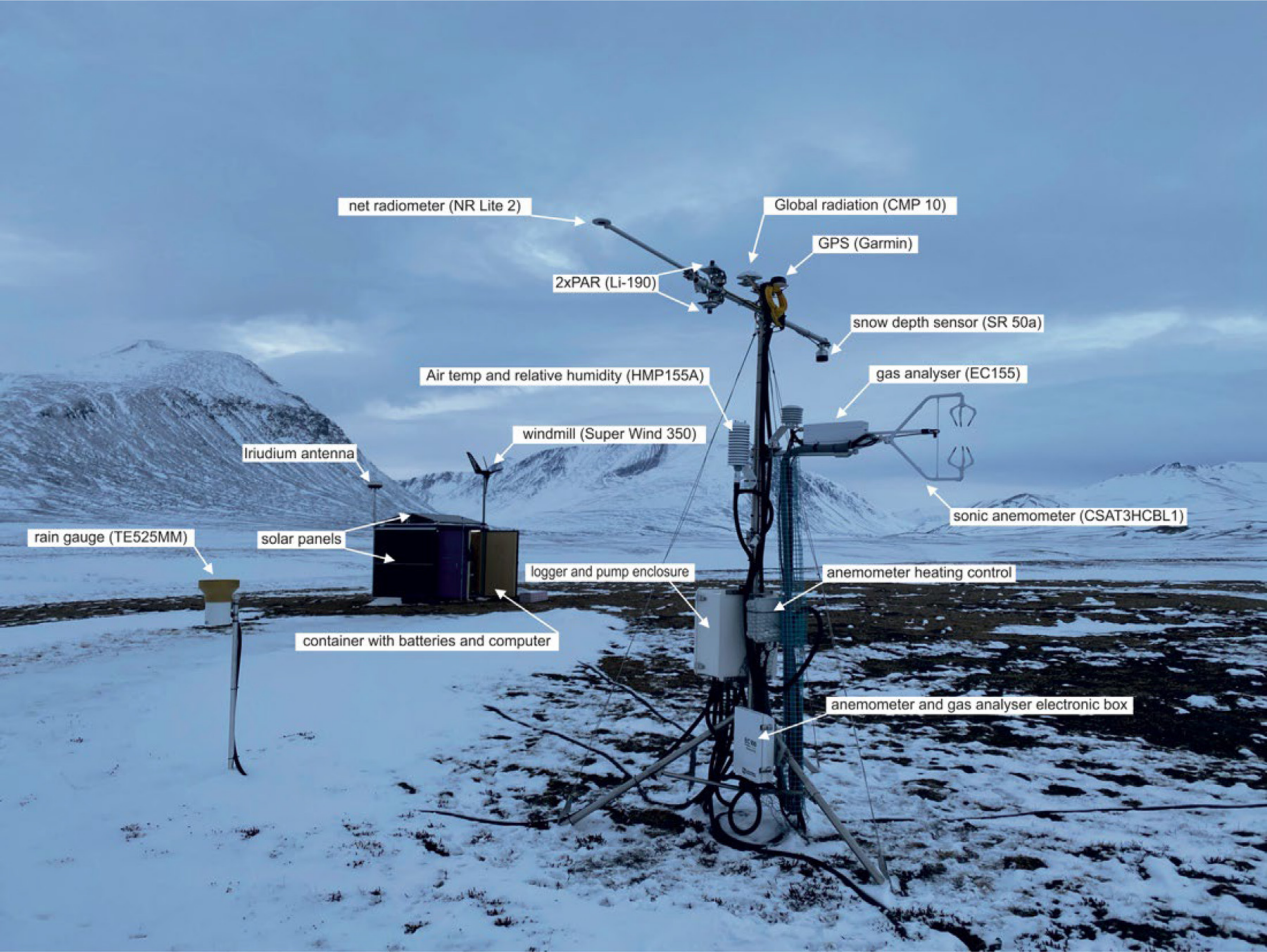
Terrestrial container eddy covariance system with meteorological sensers installed on tripod.

**Fig. 10:**
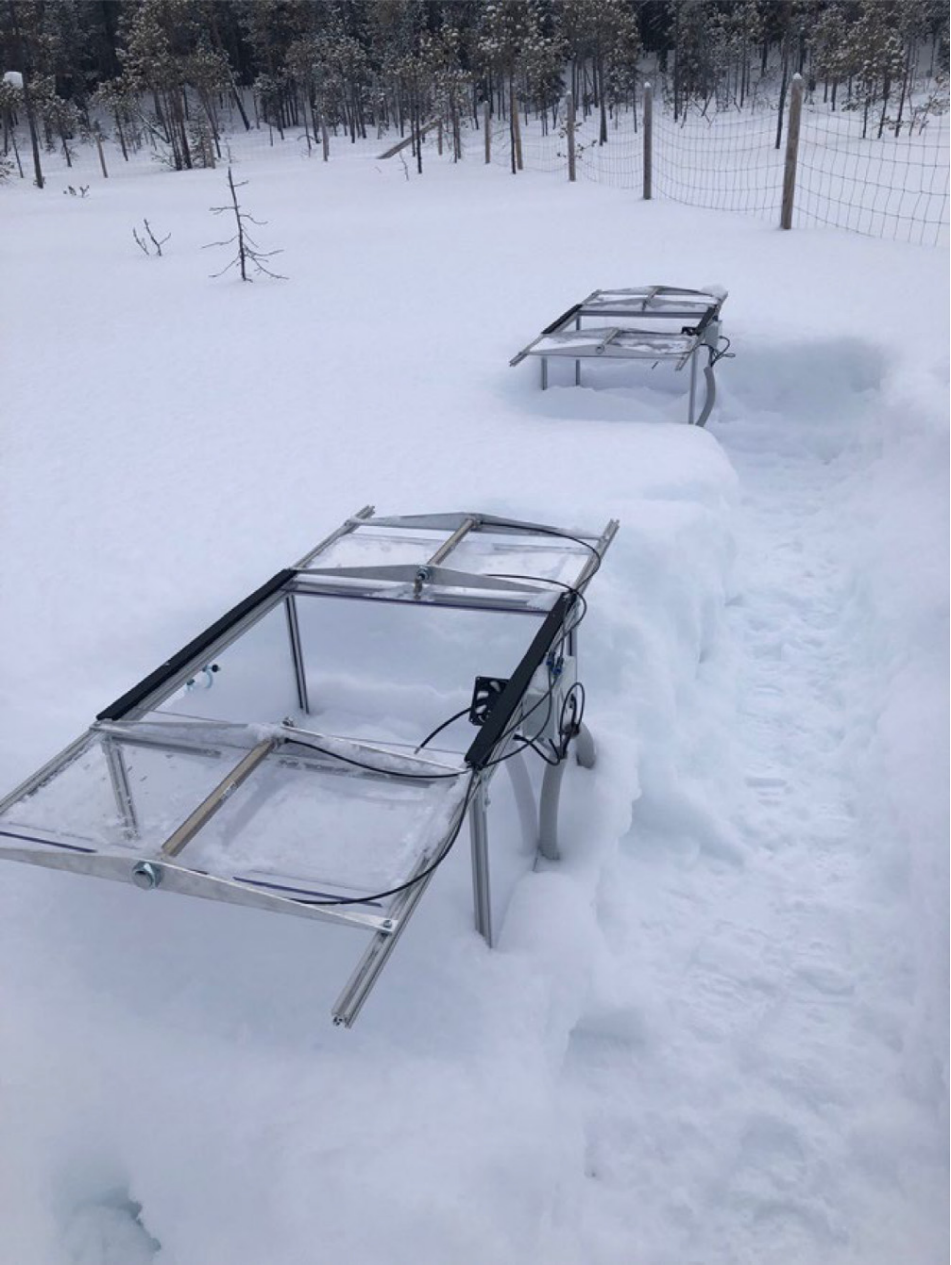
Terrestrial automatic chamber setup being tested for winter operation in Northeast Finland before deployment in Greenland.

The terrestrial component for measuring GHG gas and energy exchange is based on two different techniques: eddy covariance (EC) (Fig. 9) and a modified version of an established automatic chamber (AC) setup (Fig. 10). The EC system is based on commercial, low power consuming *CPEC306* developed by Campbell Scientific, equipped with a fast gas analyser (EC155) and sonic anemometer (CSAT3HCBL1) with a heating option installed on a stainless-steel tripod at 2.12 m height. The system operation and data collection is performed by a CR6 data logger (Campbell Sci.) which also acquires peripheral parameters such as solar radiation, air and soil parameters. Instruments measuring solar radiation are mounted on horizontal cross-boom attached to stainless steel tripod at 2.8 m height. The following spectrum of solar radiation is measured: global radiation- CMP10 pyranometer (Kipp&Zonnen), net radiation - NR-LITE2 (Kipp&Zonnen) and photosynthetically active radiation (PAR) with set of two quantum sensors –Li-190 (Li-Cor), one oriented downwards (reflected PAR) and second oriented upwards (incoming PAR). Apart from the radiation instruments the cross-boom also accommodates ultrasonic range sensor for snow depth measurements SR50 (Campbel Sci.). Air temperature and relative humidity is measured by HMP155A probe (Vaisala) - installation height 2 m. The EC tripod also accommodates a surface IR thermometer SI/111 (Apogee) – installation height 1.5 m. In addition, 5 m away from the EC mast a simple tipping bucket TE525MM (Texas) connected to CR6 logger is installed for measuring rain precipitation. The EC system is also equipped with three independent soil profiles consisting of a set of two sensors: averaging soil temperature probe TCAV (Campbel Sci.) and self-calibrating soil heat flux plate HFP01SC (Huxeflux Thermal Sensors) each. The soil profiles are distributed in a radius of 10 m from the EC setup and represent main surrounding surface biomes, barren ground, grass and shrub characteristic for the location. The EC system clock is controlled and adjusted by GPS (Garmin).

The fast EC data are collected in 10 Hz resolution (high frequency HF) and logger perform initial flux computation by averaging each 30 min of data and combining them with representative low frequency – 30 min averages (LF) of radiation, air and soil parameters acquired simultaneously. CPEC306 system is connected by ethernet cable with local computer located inside the container which collect data and sends a daily report to the server via Iridium connection.

A typical automatic chamber (AC) setup consists of 10 chambers custom-made from aluminium alloy V-Slot 2020 profiles (RatRig, Portugal) and transparent Polycarbonate (3 mm thickness on walls, 6 mm thickness on the lid). This solution allows transportation in a compact dismounted state and for easy mounting in the field. The standard chamber size is 60 × 60 cm and 50 cm high, however different sizes can be produced on the same principle. Chambers are operated by pneumatic pistons (SMC, CD85N20-250C-B) driven by a 24 V DC air compressor (STXOF7-6–24, Dankompressor, Denmark with EMD 12C 24VDC automatic drain) with working pressure 3 Bar in the pneumatic line. Sample air from the chambers is sucked through HDPE tubing (6 mm outer diameter, Eaton, Ireland) to the container where it is analysed by LI-7810 CH_4_/CO_2_/H_2_O Trace Gas Analyzer (Li-Cor Bioscience, Nebraska, USA). For the protection of the instrument, a water trap and a custom-made protection unit are set up before the analyser. The protection unit reacts on liquid water in the gas line (flood protection) and low pressure (clog protection) and switch the emergency valve to ensure the instrument keeps operational. The chamber operations are controlled by the same local computer (Lenovo T550) as the rest of the system. Depending on the distance between the container and the chambers, the communications can be realized by cables, Wi-Fi or LoRa.

### Camera system

**Fig. 11:**
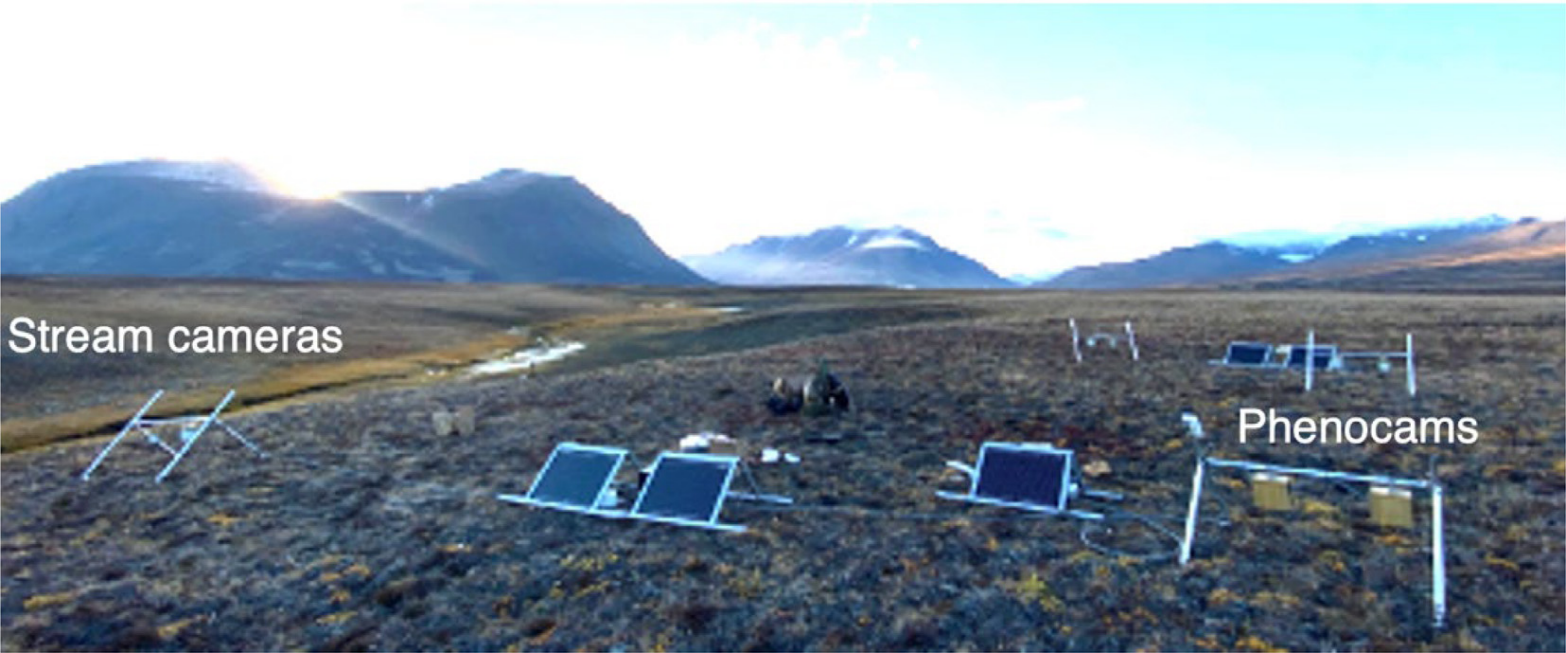
Terrestrial camera systems.

The camera system monitors plants and insects during the summer period approximately 500 m away from the terrestrial container (Zackenberg) and is supplied by its own solar power system. Two camera system are installed with a total of 8 (4 per system) nadir-facing camera designed for local-scale (<1 m^2^ extent) plant and insect monitoring. A separate powered camera system with two oblique-facing cameras is located with view over the nearby brook to monitor ice melting. A lead-acid battery with capacity of 100 Ah/12 V, a 12 V battery charger and two 60 W solar panels powers the camera system. (Fig. 11). A computer (Arduino Nano) inside a custom-built battery controller turns the power on to the Wi-Fi Bridge and camera system in the period May 15 to August 31. The Wi-Fi Bridge and camera system each consume only 12 V/7 mA during the winter period, which is a sufficiently low consumption for the battery to survive an entire year without being charged. The battery controller has a real time clock and performs timestamp logging of temperature, humidity, charge, and battery voltage every 30 min on a SD card inside the battery controller. The battery controller also ensures that the charger does not drain the battery when there is no sun during the winter period.

A 5 V power supply is turned on during the summer period to power two Raspberry Pi Zero W (Wireless) computers that each are connected to two web cameras (Logitech C922 Pro Stream 1080p). The cameras capture images in the period from 8:00 AM to 8:00 PM with a time-lapse of 60 s. Images are stored with a backup of the last 10 weeks on a Micro SD card (32 GB) inside the Raspberry Pi Zero computer. The daily captured images are also transferred every afternoon between 9:00 PM to 11:00 PM to the FTP server in the terrestrial container.

**Fig. 12:**
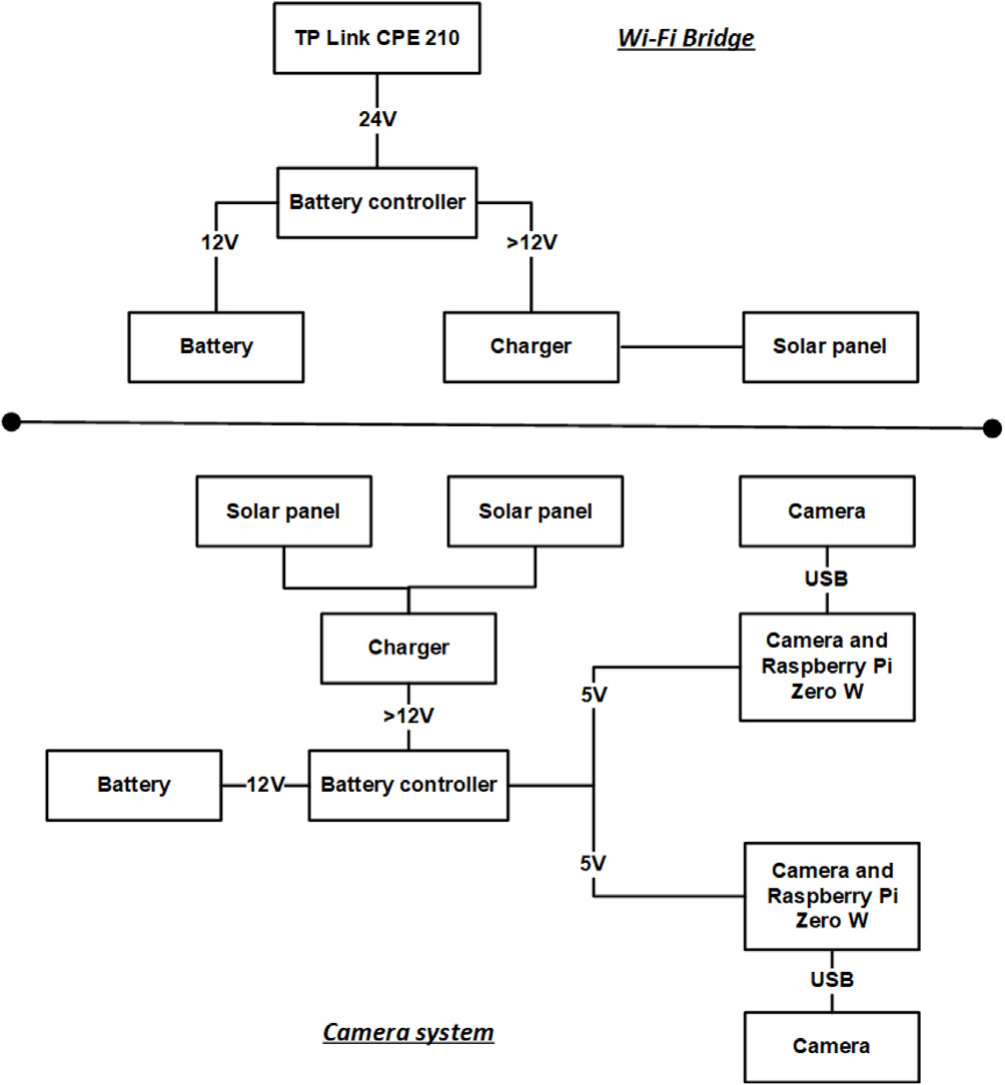
The camera system.

The Raspberry Pi Zero W computers connects to the Wi-Fi Bridge (Fig. 12) that establishes long distance connection to the terrestrial container The Wi-Fi Bridge is powered by its own solar panel, battery and charger that are similar to the devices used for the camera system. The battery controller supplies 24 V to the TP Link CPE 210 that is configured as a Wi-Fi bridge and routes communication between the camera systems and terrestrial container.

## Limnic

Data collection in streams, rivers and lakes makes up another part of the terrestrial system. The measurements are divided into two parts: 1) whole-year data collection, and 2) additional data collection in open-water season. Parameters included in whole-year data collection are determined by which available sensors are able to withstand being frozen into water. These include water temperature (TinyTag; Gemini data loggers UK), light intensity (HOBO instruments, USA), specific conductivity (HOBO instruments, US), dissolved oxygen (DO; miniDOT, PME instruments, USA) and level logger transducer (Solinst, Canada). Freezing resistance of HOBO, PME and Solinst instruments are currently being tested.

At selected sites we include measurements of water dissolved organic carbon (DOC), turbidity and NO_3_ by multi-sensor sondes (e.g. YSI, USA or S:CAN, Austria) during the open-water season. These multi-sensor sondes are unlikely to withstand in-freezing in the water during winter, and this data collection therefore requires at least two annual visits to the monitoring site. No lake or large river site is yet included in the monitoring.

In addition to measuring water chemistry, we install two cameras near the stream with one facing over the stream reach and one facing vertically into the stream water. Power supply and data transmission to and from camera and multi-sensor sondes is like the systems described for the plant and insect camera systems above.

## Ocean

### Ocean cabled observatory

**Fig. 13:**
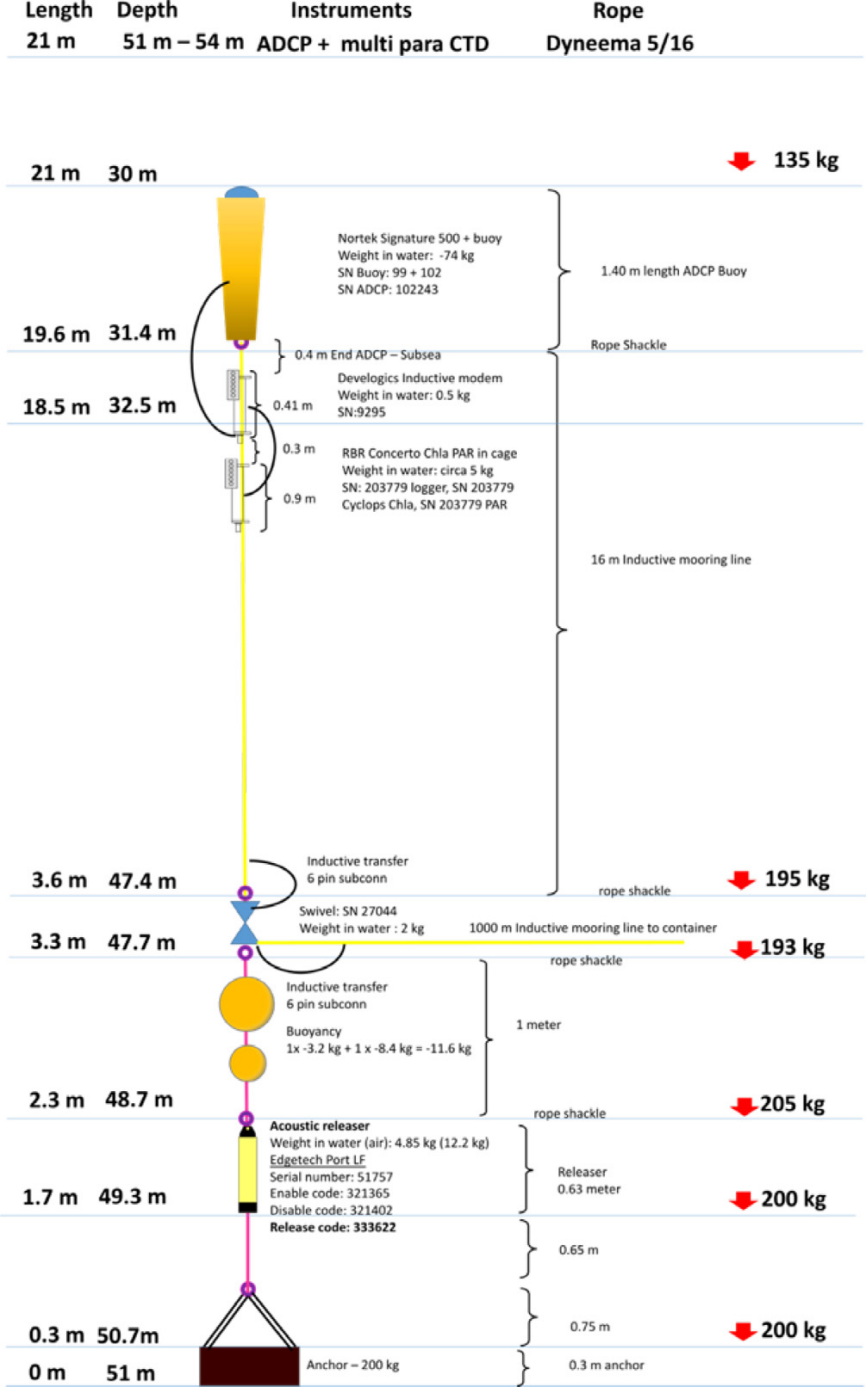
Ocean cable observatory.

The ocean cabled observatory consists of an inductive link and sensors, an anchor, acoustic release, dyneema lines and buoyancy floats. The inductive link has a topside and subsea inductive modem (Develogic) interlinked with an inductive cable (horizontal length: 1 km, vertical: 16 m) and a swivel. The interfaced sensors, connected to the subsea inductive modem are a multiparameter ocean sensor and an acoustic doppler current profiler (ADCP) (Fig. 13).

The subsea unit consist of a data logger with inductive modem, internal battery pack (12 D cell lithium), magnetic power switch with status indicator and battery monitoring, a safety valve and 3 ports for sensors interfacing. The hard anodized aluminium housing of the subsea unit is rated for deployments up to 500 m depth.

The topside consists of a data logger with inductive communication interface and backup iridium modem. The combination of the topside and subsea unit allows for bidirectional transmission of data and configuration commands.

The inductive link consists of a 4 mm steel cable extruded to 6 mm with yellow polypropylene (PP) plastic and a breaking strength 9.5kN. The cable ends are terminated by titanium terminals that function as seawater electrodes for the inductive link. The vertical part of the cable is linked with the horizontal line on the seafloor via a titanium swivel with conductive feed through. The swivel has a 15 kN safe operating load and is suitable for both inductive and wired mooring setup.

Various instrumentation can be attached to the inductive link. In the present setup we selected a Nortek Signature 500 kHz ADCP instrument deployed in a protective buoy for telemetering hourly averaged velocity profiles and for 12 hourly derived ice keel and drift measurements. The RBRconcerto CTD instrument was selected for conductivity, temperature, and depth (pressure) recordings and it was furthermore equipped with a Turner fluorometer for Chlorophyll determination and a LI-COR PAR sensor for irradiance measurements. Both sensors are streaming serial data to the subsea unit which forwards the data to the topside unit where it is stored until the topside unit is interrogated by the container telemetry software for data submission to the servers.

The mooring setup further consists out of a 200 kg steel H-bar connected to an acoustic release (Edgetech Port LF) and 2 trawler beats (1 × 3.2 kg + 1 × 8.4 kg) which functions as buoyancy. The nodes are connected via dyneema lines of 7.95 mm (5/16 in.).

## Deployment of cable 1 km out to 50 m depth

**Fig. 14:**
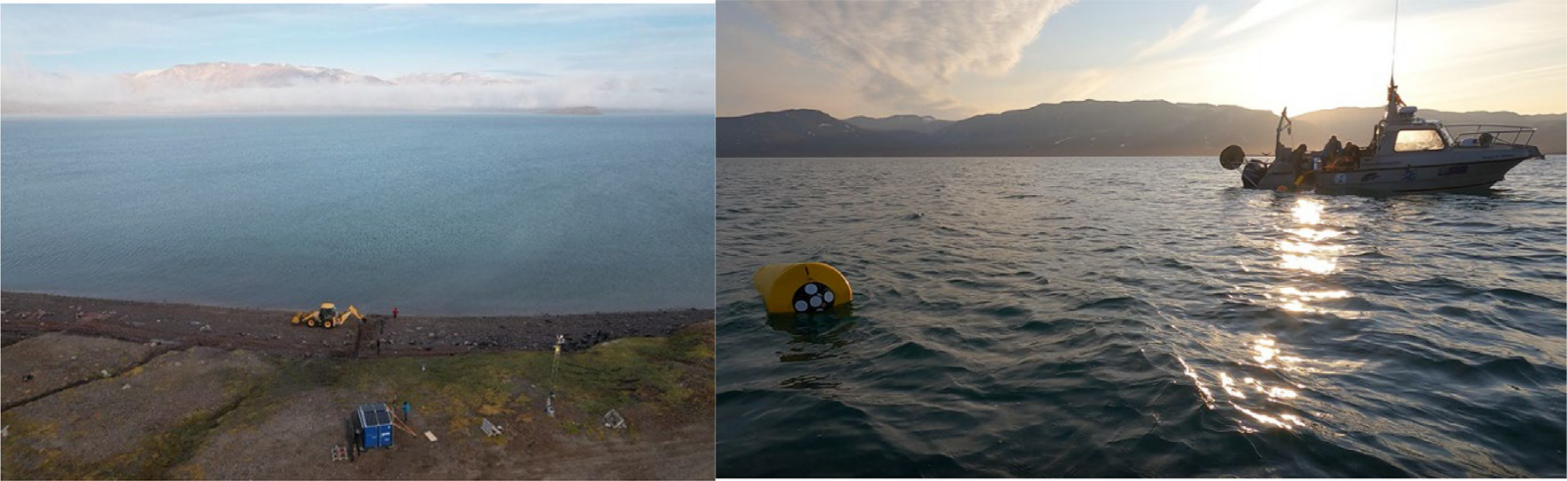
(Left) Digging a trench to protect the sub-sea cable from ice scour and disturbance at the shoreline. A channel was dug during lowest neap tide. (Right) installation of the inductive cable to the mooring position (1 km off coast) and deployment of the mooring with a small support vessel.

The cabled observatory was installed in a seasonally ice-covered fjord (Young Sound in NE Greenland, 74°21 N, 20°21 W) (Fig. 14). To protect the cable against scouring effect of sea ice and icebergs the first 25 m of the cable was protected by polyethylene (PE) pipes welded together via electrolysis. During lowest low water, a trench was dug to bury the PE pipes and cable in the sediment to further prevent the effect of ice on shore landing of the cable. We were fortunate to borrow a digger from the Sirius Dog Sled Patrol, but the work can also be done by hand, making it possible to install units in remote areas with helicopter access only. The spool with 1 km cable was loaded on a 6 m support vessel and unwinded to the deployment area, where the mooring was anchored on the seafloor. The releaser attached to the anchor allow for an acoustic release of the mooring line when the mooring is to be inspected or instruments replaced each year or second year with new calibrated sensors and charged batteries.

## Data storage and transmission

**Fig. 15:**
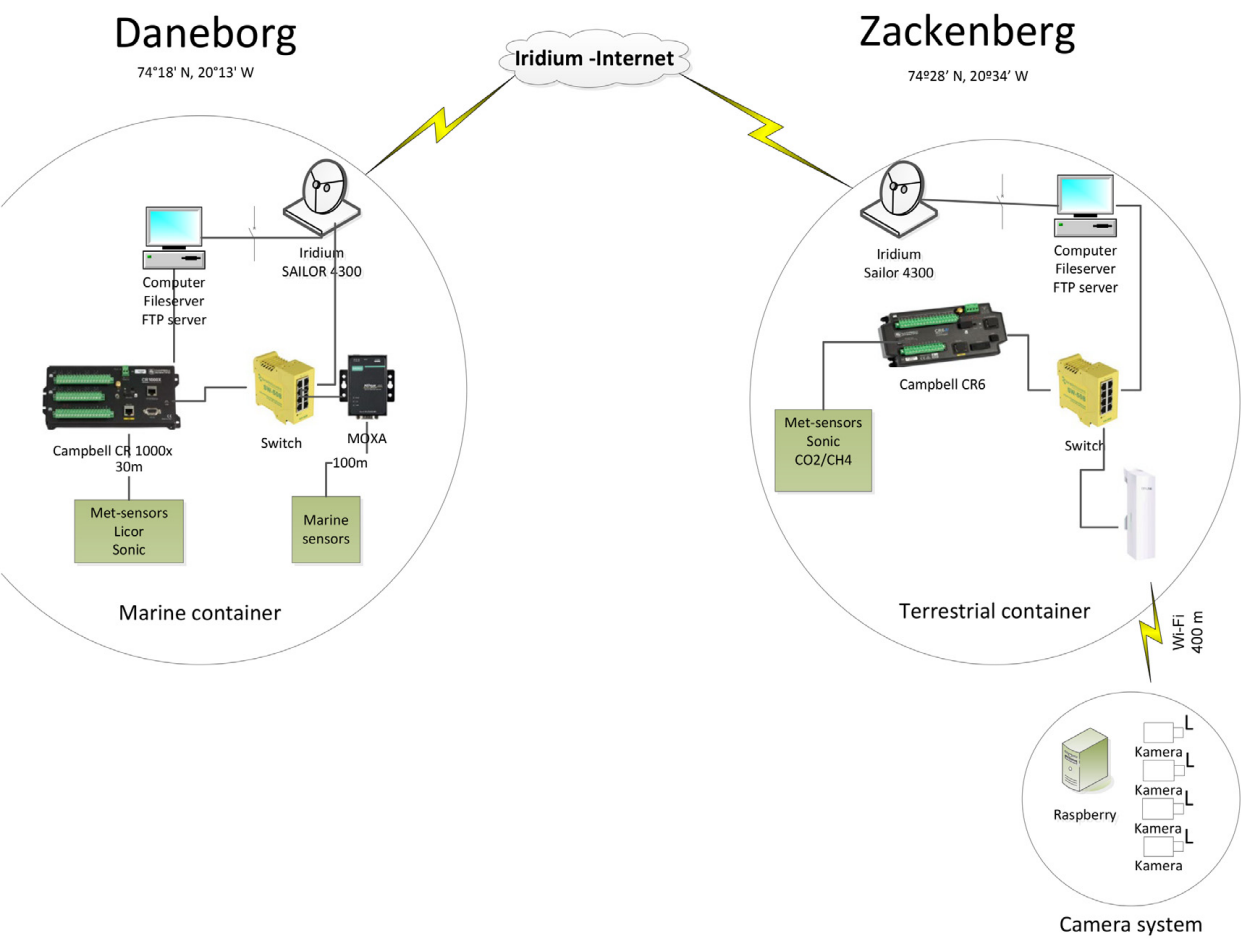
Data acquisition and satellite system.

The atmospheric and terrestrial data from the ARC-MO units are collected directly on a CR6 datalogger equipped with 16 GB industrial grade microSD memory card. The ocean data is stored on the topside unit. Daily, all data are synchronized with a local computer (Lenovo T 550) located inside the container (Fig. 15). HF data are collected via ftp protocol directly from logger memory card and LF data are synchronized with the use of LoggerNet software with a build-in schedule collection feature. All data are saved and stored on the internal HDD of the Lenovo T550 computer. The local computer initiates Iridium connection on daily basis and transmit single a LF data file to data server.

### Server

A Lenovo T 550 computer is used for data handling, FTP- and application server in both containers (Marine, Terrestrial). This model has standard operating specifications suitable for work in extreme environments (low temperature, high humidity, low power consumption). It is also designed to restart after an optionally power failure. Both average data and high frequency data from the data loggers and camera system is stored on a 2 Tb SSD hard disk.

### Data transmission

In both containers, the server runs a scheduled job for transmitting data by the Iridium satellite system (Iridium Sailor 4300) (Fig. 15). The job turns on a relay to power up the Iridium modem, compress data files, waits until an internet connection is established, then transmit data by FTP. The connection is then closed, and the modem powered off. The data files are currently transmitted to a FTP server at Aarhus University. If the connection is not established and/or data files are not transmitted successfully, the computer will retry to send the data files later. It is also an option to access the station by an external FTP connection and download data or upload new applications or setup files (via static IP address and using port forwarding). Only averaged result files and status files are sent, currently below 100 Kb each day. The max transfer speed is 172 Kb/s. Raw files (high frequency data etc.) and pictures from the camera system are saved on the hard disk (and on the Campbell data loggers as long as space is available). To keep telemetry costs acceptable, these files need to be collected when visiting the station.

### Wi-Fi connection

Between the two containers, Ubiquiti Rockets (Prism 5AC 5 GHz) and Ubiquiti airMax disk antennas establish a data link via a long range Wi-Fi connection (Fig. 15). The distance between the two containers is 25 km. Moreover, it is possible to transmit data within the stations. More specifically, between the terrestrial container (Zackenberg) and the camera system and the limnic sites. There, a TP Link CPE 210 establishes the Wi-Fi connection. We selected a 2.4 GHz system, because the Raspberry Pi computers also connect by Wi-Fi, and do not support 5 GHz. The camera system is only running in the summertime (from May 1. to August 31.). For the rest of the year, the Wi-Fi system is turned off.

## Data access

**Fig. 16:**
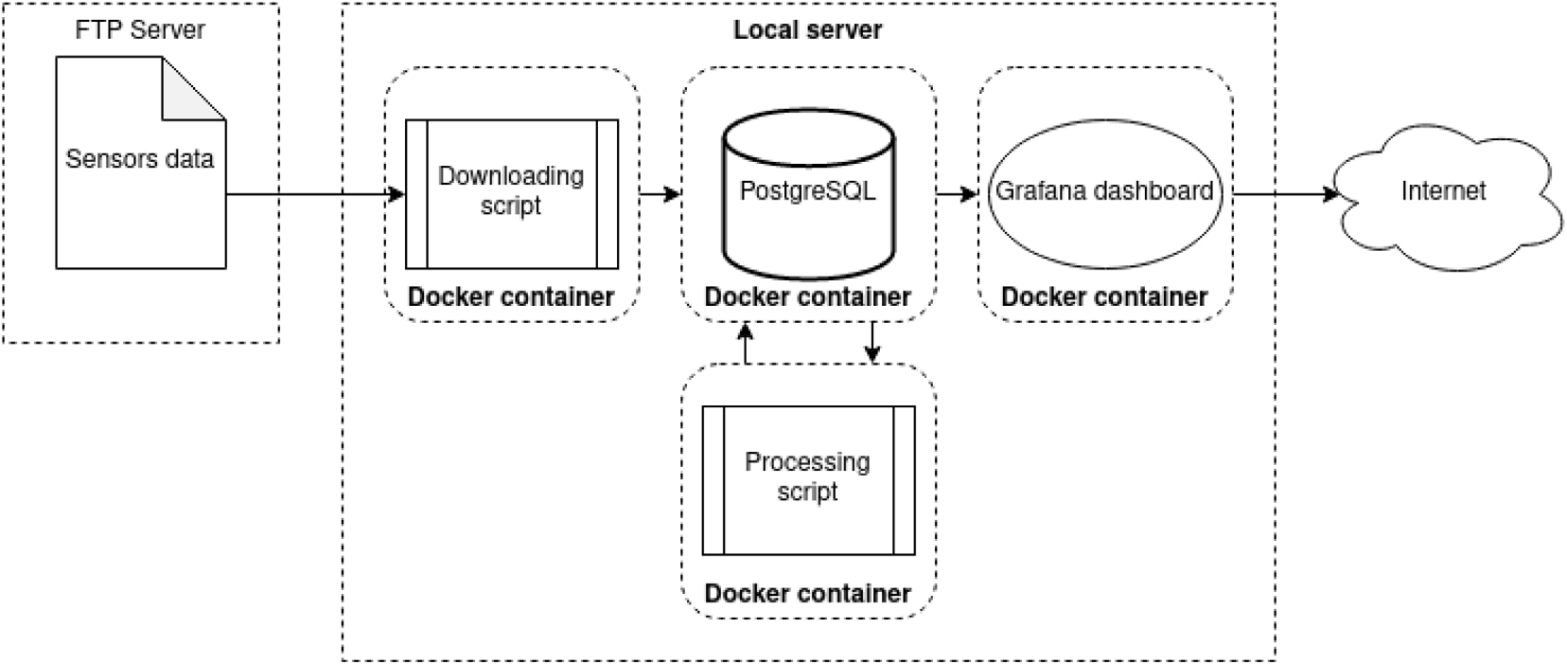
The diagram of the data flow from FTP server to Grafana dashboard.

The data stored on the FTP server is automatically transferred via a downloading script running on a local server to a local PostgreSQL database (Fig. 16). There, a processing script generates derived values from the measurements to calculate parameters such as sea ice thickness and status of the system and system components. The database is then linked with a Grafana dashboard to visualize selected measurements, derived values, and health parameters from the system. Each link of this chain is deployed locally on the server via a Docker container, which allows the installation to be reproducible and scalable. The coding is done in the Python programming environment, which makes it easy to read and reusable by a large community of users.

**Fig. 17:**
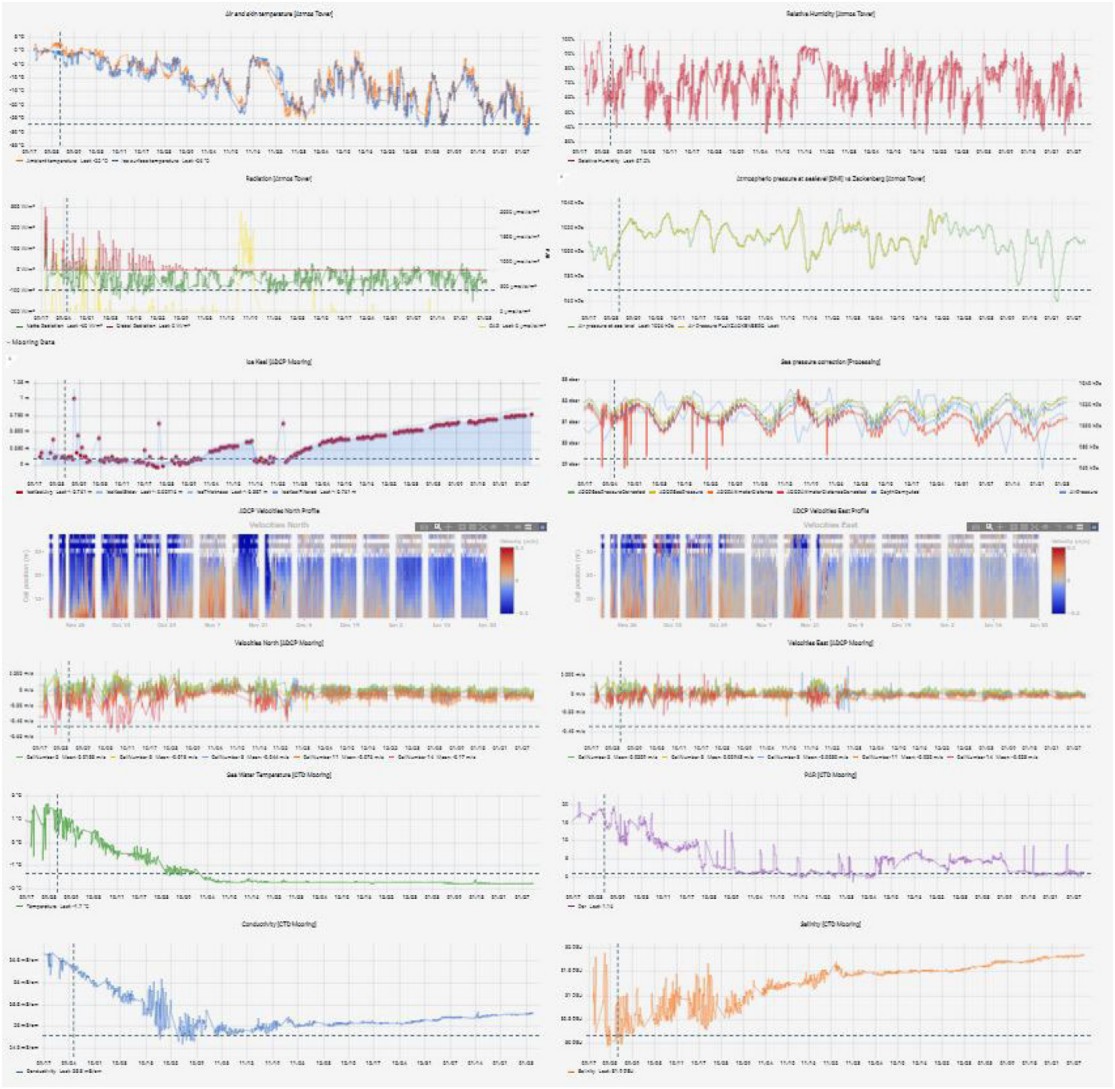
Grafana dashboard example with indication displaying information of the container at Daneborg in NE Greenland.

The Grafana dashboard allows to dynamically change the time range of visualization and to have an overview of all the available data (Fig. 17**)**. We use a combination of standard plotting tools and custom plugins (plotly, Grafana Labs) in the Grafana dashboard to display both time series data and profile data of the acoustic doppler current profiler (ADCP). Visual links between different data plots can be made through a duplicated cursor shown on all the graphs. Furthermore, Grafana allows to generate alerts based on the received data, which allows users to be notified by changing status of the system or by environmental changes detected by the sensors.

All displayed data (raw, filtered and derived data) can be downloaded directly through the application in csv format. This comprises both the raw data generated by the sensors, the filtered data and the derived data. Data tiles can be shared via links, snapshots and embedded via an automatically generated html code.

The data is available on our Greenland Integrated Observing System (GIOS) homepage https://gios.org.

## Price

**Fig. 18:**
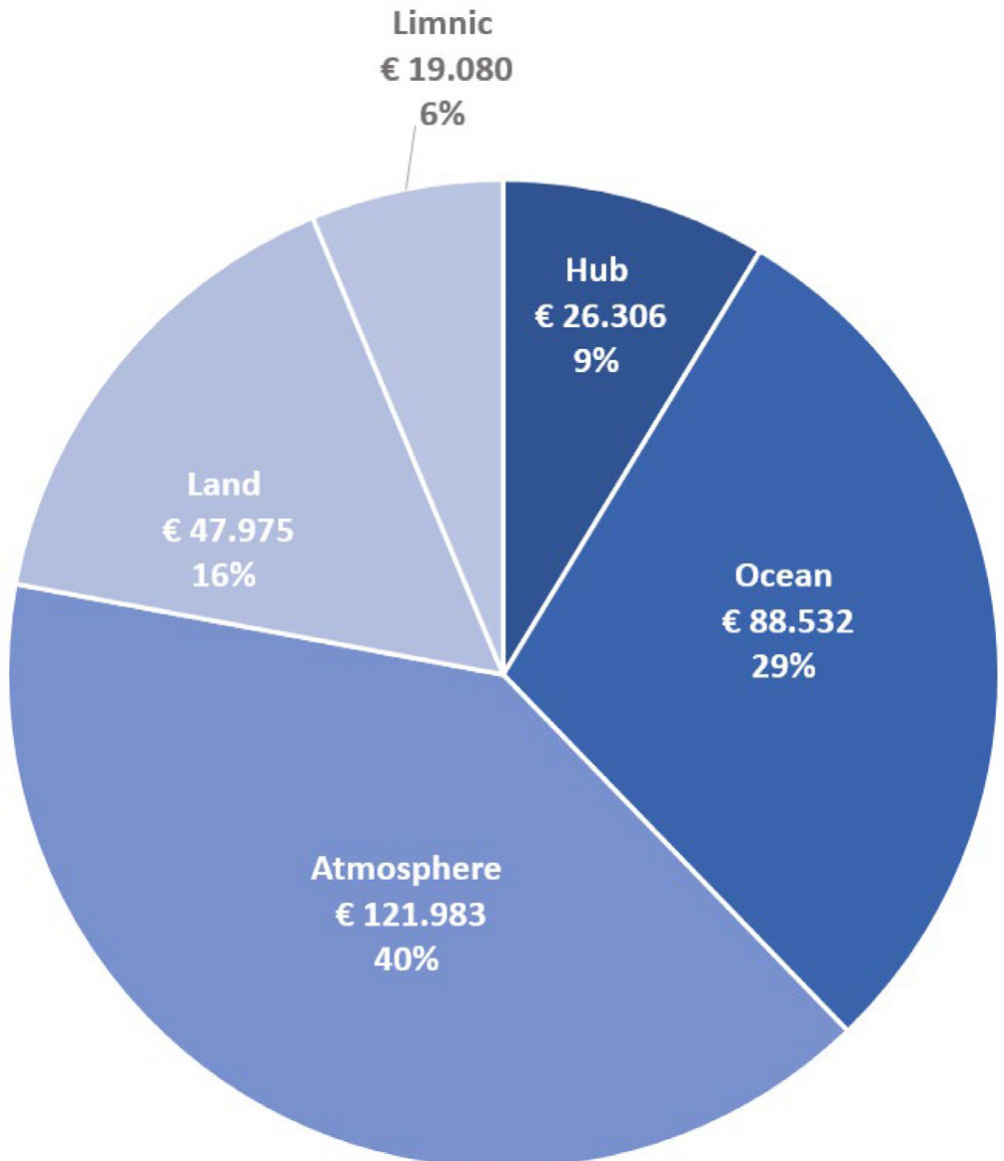
Price overview of ARC-MO showing the cost of the central hub, atmospheric, land and ocean instruments.

The cost of the various components of ARC-MO is presented in Fig. 18. The central hub containing solar panel, windmill, batteries, wiring system and server systems accounts for 9 % of the total cost, the other units, e.g. atmosphere, land, limnic and ocean can be combined as desired. The cost for a full system covering atmosphere, land and ocean is 304.000 €. A detailed Bill Of Material (BOM) file can be found in design file (BOM.xlsx).

## Design files summary

**Fig. 19:**
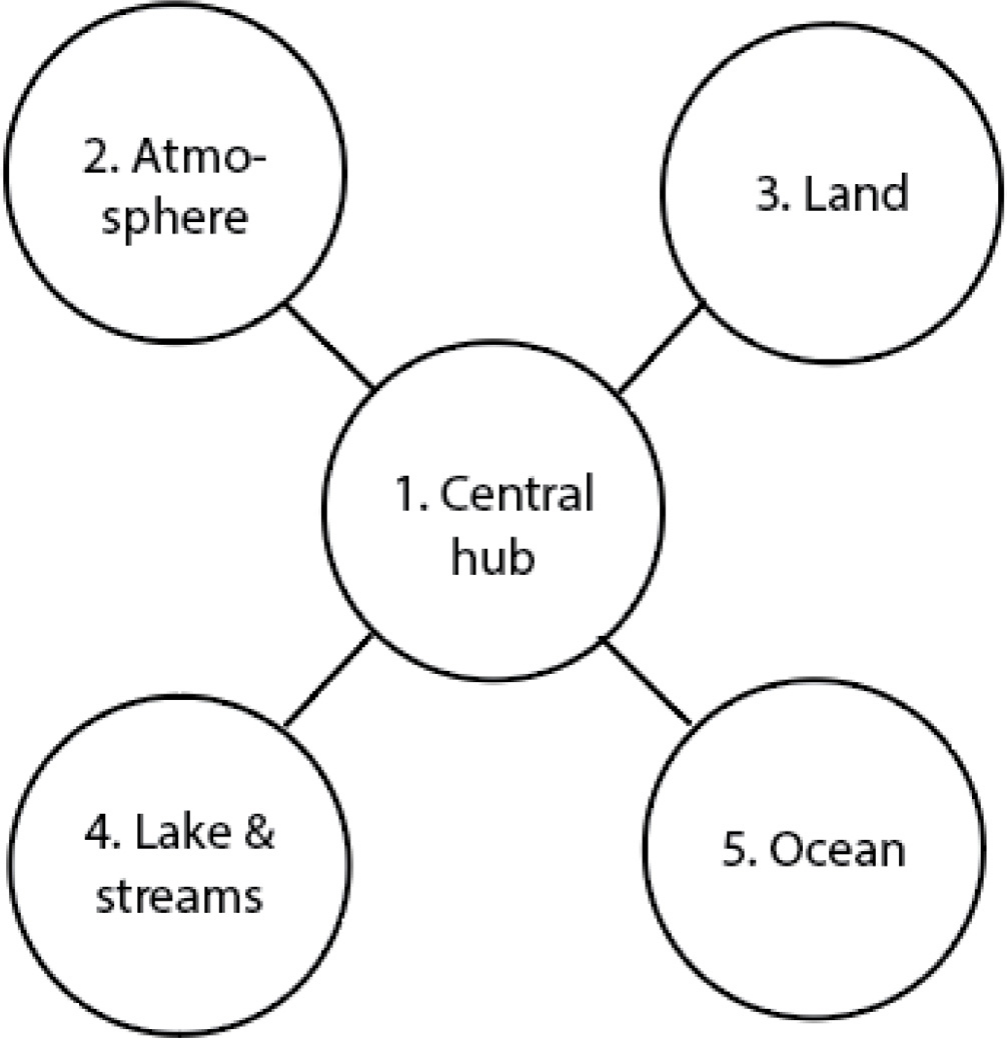
Overall view of the ARC-MO schematic design of system.

The design files for the ARC-MO are organized into five different components: (1) the central hub connecting the different measuring units in the (2) atmosphere, (3) on land, (4) lakes & streams and (5) the ocean (Fig. 19).

The design files can be found at: https://doi.org/10.17632/5gfmy98kcj.2.

**Table:** 

Design file name	File type	Open source license	Location of the file
BOM.xlsx	Excel	Contains detailed costs of materials	https://doi.org/10.17632/5gfmy98kcj.2
*1. Central hub*			
1.01_container_setup	PDF	Contains information on the container unit	https://doi.org/10.17632/5gfmy98kcj.2
1.02_communication	PDF	Contains information on the WiFy and satellite communication	https://doi.org/10.17632/5gfmy98kcj.2
1.03_power	PDF	Contains information on the power and battery system	https://doi.org/10.17632/5gfmy98kcj.2
*2. Atmosphere*			
2.01_meteorology	PDF	Contains information on the meteorology mast and sensors	https://doi.org/10.17632/5gfmy98kcj.2
*3. Land*			
3.01_soil	PDF	Contains information on the soil sensors	https://doi.org/10.17632/5gfmy98kcj.2
3.02_precipitation	PDF	Contains information on precipitation (rain and snow) gauges	https://doi.org/10.17632/5gfmy98kcj.2
3.03_fluxes	PDF	Contains information eddy and chamber flux systems	https://doi.org/10.17632/5gfmy98kcj.2
3.04_photos	PDF	Contains information on time lapse camera systems	https://doi.org/10.17632/5gfmy98kcj.2
*4. Lake & streams*			
4.01_sensor array	PDF	Contains information on limnic sensor systems	https://doi.org/10.17632/5gfmy98kcj.2
*5. Ocean*			
5.01_cable observatory	PDF	Contains information on ocean cabled sensor systems	https://doi.org/10.17632/5gfmy98kcj.2

## Bill of materials

### Bill of materials summary

**Table:** 

Designator	Component	Number	Cost per unit - EUR	Total cost -EUR
	*Name of Component 1*	*Number of units*	*Cost per unit and the currency used*	*Total cost and the currency used*
1.01	Container	1	5.766 €	5.766 €
1.02	Communication	1	5.515 €	5.515 €
1.03	Power	1	15.025 €	15.025 €
2.01	Meteorology	1	121.983 €	121.983 €
3.01	Land, soil	1	3.938 €	3.938 €
3.02	Land, precipitation	1	1.267 €	1.267 €
3.03	Land, fluxes	1	34.567 €	34.567 €
3.04	Land, camera system	1	8.203 €	8.203 €
4.01	Limnic observations	1	19.080 €	19.080 €
5.01	Ocean cabled observatory	1	88.532 €	88.532 €
Total			303.877€	303.877€

## Build instructions

The building instructions for the ARC-MO is organized into 5 components according to Fig. 19.

1. Central hub

1.01. Container setup

• (1.01_01) Make a foundation for the container of 4 × 200 L half oil drums and fill them with sand, stones and concrete. Dig drums half into the ground. Place the container on the foundation, level it and make sure it is lifted above ground ensure meltwater can escape during snowmelt and not get into the container.
• (1.01_02) Move or sling the 6-foot insulated aluminium container with truck or helicopter to the deployment site.
• (1.01_03) Install two anchors (same as foundation), one to secure the inductive cable to the ocean mooring and the second to keep the container in place under northerly storms.
• (1.01_04) Two solar panels are mounted one the side of the container pointing in a southernly direction, and two panels are mounted in top of the container in an angle of 15 deg.
• (1.01_05) Mount a windmill pole (ø60 mm) onto the side of container with three bolts, top, mid and bottom. The pole foot (bottom) is mounted into the concreate foundation.
• (1.01_06) Attach the windmill generator unit to the pole.
• (1.01_07) Insert cables from solar panels, windmill and instruments through a vent on the back of the container. Seal the vent with foam to prevent snow blowing into the container.
• (1.01_08) To protect all outside cables from wildlife destruction, encapsulate all cables in protective piping and attach them to the wall of the container.

1. 02. Communication

· (1.02_01) Mount the Iridium antenna on the pole (ø60 mm, 3 m long). On the container mount the TP-Link router as well on the pole aligned with the TP-Link router on the camera system. Attach it with a clamp on the top. Make sure the bottom is resting on the ground. Install protection for antenna cables, power cable, Ethernet cables and route them inside container thought ventilation openings.
· (1.02_02) Mount the Rocket dish and station on the pole (ø60 mm, 5 m long). Attach it with clamp on the top and near the ground. Make sure the bottom is resting on the ground. Install protection for Ethernet cable and power cable, route them inside the container though ventilation openings, and attach the Ethernet cable in the switch on the wall. Careful align the dish between the containers.
· (1.02_03). Place the server and the USB relay in an isolated aluminium box inside the container. Place the Iridium router outside the box. Connect the Iridium router and server with Ethernet cables to the Ethernet switch on the wall of the container. Connect power by 19.5 V to the computer. Connect power (24 V) for the iridium router going through the relay.
· (1.02_04) Install a 16 Gb micro industrial SD Card in the data logger. Place the data logger and connections for sensors in a box on the mast. On the Atmospheric, use an Ethernet switch to collect Ethernet cables (the distance between the mast and container must not exceed 100 m). Connect an Ethernet cable and route it together power cables into the container thought ventilation openings. Encapsulate all cables going from the mast into the container in protective piping. Inside the container attach the power cables and connect the Ethernet cable in the switch on the wall.

1.03. Power

• (1.03_01) Batteries are placed in the transport cage and transported to the container unit. Remove the sidewalls from the battery cage. Organize batteries in 2 layers with a wood placeholder between the batteries for the cables. The top plate is replaced to protect the batteries and serve as a working bench for the server. Connect batteries to the wiring system upon arrival at the field location.

2. Atmosphere

2.01. Mast set up

· (2.01_1) Find a solid flat ground of a suitable size or make a foundation both depending on the surface for the mast 45 cm triangular guyed mast incl. bottom plate.
· Install three spears or eyebolts of a suitable size in the ground of each of the corners of the mast depending on the surface in a distance away from the mast according to standard. (approx. same distance away from the mast as the distance from the ground to the point where the guy wires are attached to the mast.). We have been using a detachment point approx. ⅔ up the tower.
· Attach the three 6–8 mm guy wire to the mast, one in each corner of the mast, using suitable Eureka wire locks (2.01_02) (or other brands).
· Raise the mast on the flat ground or on the foundation.
· Fasten each of the wires attached to the corners of the mast to a turnbuckle using suitable Euraka wire locks, and attach the turnbuckle to each of the spears/eyebolts
· Tighten the wires so the mast is stable (do not over tighten the wires), and safe to climb.
· Drill 4 holes in a straight line with a distance of 50 cm, starting approx. 5 cm from one end, and one hole in the opposite end in two 3 m long, ø50 mm aluminum booms in order to be able to install various types of equipment.
· Install two mast clamps (2.01_03) for each of the aluminum booms in the chosen height, and level them horizontally.
· One boom should point towards the water (mounted on the side of the mast, which is 90 deg of the coastline), and one should be pointing against the prevailing wind direction mounted on the side of the mast facing the water.
· Make sure the pre-drilled holes in the booms are pointing towards the water for one boom and towards the prevailing wind direction for the other one.

2.02. Meteorology

· (2.02_2 & 2.02_3) Install the ultrasonic anemometer at the hole at the end of the boom (hole 1) pointing towards the water.
· Install the ultrasonic anemometer electronics box directly to the mast an approximately 3.5 m height and connect the sensor.
· Install a PAR sensor at the end pointing opposite the ultrasonic anemometer.
· Mount the skin temperature sensor on the “LI-7700 CH_4_ sensor boom” just outside the mast and point the skin temperature sensor at a point always a of the coast the in the water. Opposite the LI-7700 CH_4_ sensor the global radiation sensor must be installed on the boom, and the net radiation sensor must be installed further away on its own boom.
· The temperature/rel. humidity probe can be installed directly to the mast a little below the boom arrangement.

2.03. Climate gases

· (2.02_02 & 2.02_03) Install a LI-7200 CO2 sensor in hole number 3 at the “ultrasonic anemometer boom” with a supporting pole for the heated inlet in hole number 2.
· Install the LI-7200–101 pump module directly to the mast at approximately 3.5 m height and connect the sensor.
· Install the LI-7550 AIU next to the LI-7200–101 pump module and connect the instrument.
· Install a LI-7700 CH_4_ sensor at the end of the boom facing the prevailing wind direction. Make sure the LI-7700 CH_4_ sensor is “behind” the ultrasonic anemometer, by moving the boom with the instrument towards the mast.

2.04. General

· (2.04_01 & 2.04_02) Install the data acquisition (DAQ) cabinet just below the LI-7550 AIU and LI-7200–101.
· Connect all instruments to the DAQ cabinet and connect the cabinet to the container.
· All instruments are connected using Burndy plugs, where female pins are mounted in the cabinet (2.04_01), and the instrument cables are fitted with male pins (2.04_2).
· All cables and the tubing for all instruments must be fastened closely to the booms and down one leg of the mast.
· Excessive cables must be coil up and fastened to the mast at approx. 3 m height.
· All cables running to/from the container are protected by a heavy cable conduct on the ground and a ø50 mm aluminum boom section from the ground to the DAQ cabinet due to wildlife.
· We are using vinyl electrical tape (Scotch Super 88) for fastening cables and tubing.

3. Land

3.01. Soil

(3.01_01) Make three ∼ 20 cm deep soil openings with shovel (in our case ∼ 9 m away from the EC tower – maximal distance allowed by sensor cable length). Insert soil temperature sensors at −0.5, −5.5, −10.5 and −15.5 cm depth in one vertical line and insert soil heat flux plate sensor into suitable soil pocket at the depth of −16.0 cm (important to insert the probe plate in correct orientation indicated on the sensor). Fill the opening with extruded soil making sure buried sensors have proper contact with soil. In areas where wildlife interaction likelihood is high it is recommended to protect the cables with robust cable conducts. Connect sensors to CR6 logger.

3.02. Precipitation

(3.02_01) Mount rain gauge on the separate 1 m tall pole in some distance from the mast (in our case ∼ 7 m West). Since this sensor is relatively light, a solution with simple pole inserted directly into the ground was sufficient in our case. In areas where wildlife interaction likelihood is high it is recommended to protect the cables with robust cable conducts.

3.03. Fluxes air meteorology and radiation sensors

• (3.03_01) Unfold the stainless steel tripod some distance from the container (in our case 20 m East – limited by the length of both power and ethernet cable). Adjust tripod legs to make sure the central tripod pole is vertical. Mount EC system enclosures (logger with pump, anemometer electronic box - EC100, as well as anemometer heating control unit) according to manufacturer instructions. Install GPS and global radiation sensor at the top of the centre pole with use of provided mounts. Install cross boom for other radiation sensors (should be installed as high as possible and oriented along North-South direction). On southern end of radiation cross boom install net radiometer (suitable mount is provided by manufacturer) as well as 2xPAR sensors. On the northern end of radiation sensor install snow range sensor. Install anemometer and gas analyser cross boom. To minimize the construction influence on eddy covariance measurements anemometer and gas analyser cross boom should be oriented perpendicular to main wind direction specific for the site (in our case 86°, N for main wind direction N—S). Finally, install air temperature and surface temperature sensors on the main pole of the tripod with mounts provided by manufacturer.

3.04. Camera system for recording nadir photos at high temporal frequency

• (3.04_1) This sensor system consists of power supply units, camera units and a WiFi bridge connecting the sensors to the terrestrial container. Camera units are mounted on a rack built from aluminium tubes assembled with K clamps. They must be positioned with a distance of *c.* 30 cm above the plants to record. The camera units connect to the Wi-Fi bridge, which again connects to the server in the container. The power supply units consist of two solar panels, a charge regulator, battery and battery controller (Fig. 12). The two solar panels should be connected in parallel to the input of the charge regulator. The output of the charge regulator is connected to the battery controller. The battery controller is connected to the lead-acid 12 V battery and supplies 5 V to the Raspberry Pi’s with cameras. The power supply to either the camera units or the WiFi bridge is shut down by battery controllers during the winter period to ensure efficient power management, a period when solar panels are unable to deliver power to the batteries. Cables connecting power units and camera units as well as the WiFi bridge are protected by PVC tubing.

4. Rivers & streams

4.01. Sensor array

• (4.01_01) Sensors planned to be used in entire year data collection.
• (4.01_02) Sonde deployed in open water season for measurements of additional parameters.
• (4.01_03) Deployment of sensors and sondes in the first GIOS stream.
• (4.01_04) Camera facing the stream reach, where sensors are deployed.

5. Ocean

5.01. Cabled observatory

• (5.01_01) At the shore, the inductive steel cable is inserted into a PE pipe (ø11 cm), dug into the ground during neap tide from the waterline to the container unit to protect it from ice and from destruction by wildlife.
• (5.01_02) The spool with 1 km cable is unwinded from the shore to the deployment area.
• (5.01_03) At location, all mooring hardware, previously prepared and loaded on the support vessel, are connected to the cable, and anchored on the seafloor.
• (5.01_4) Finalize protection of the subsea cable all the way into the container through the snow proof valve on the back of the container and connect it to the topside unit.

## Operation instructions

### Central hub

Safety instructions and maintenance of the ARC-MO (central hub) system when visiting in the field:

1. Check for loose cables and damage to the solar panels.

2. Check the mounting systems of solar panels and wind turbine are firmly held by mounting clamps and mounting rails/tube are firmly attached to container sides and roof.

3. Check wind turbine has no abnormal noises and vibrations.

4. Battery: Perform visual inspection (wear safety goggles):

a. If there is corrosion on the battery terminals, clean them.

b. If you have noticed any damage to the case, it is best to replace the battery.

## Battery check, measure the battery voltage using a volt / multimeter

a. Below 18 V, the battery is in critical condition.

b. If the battery voltage is lower than 22.5 V, it means that the battery is discharged.

c. A voltage of<25.2 V means that the battery is<50 % charged.

d. Voltages between 26.4 and 27.36 V are considered 100 % charged.

e. Check all battery voltages; these must be within 1 % of each other.

**Fig. 20:**
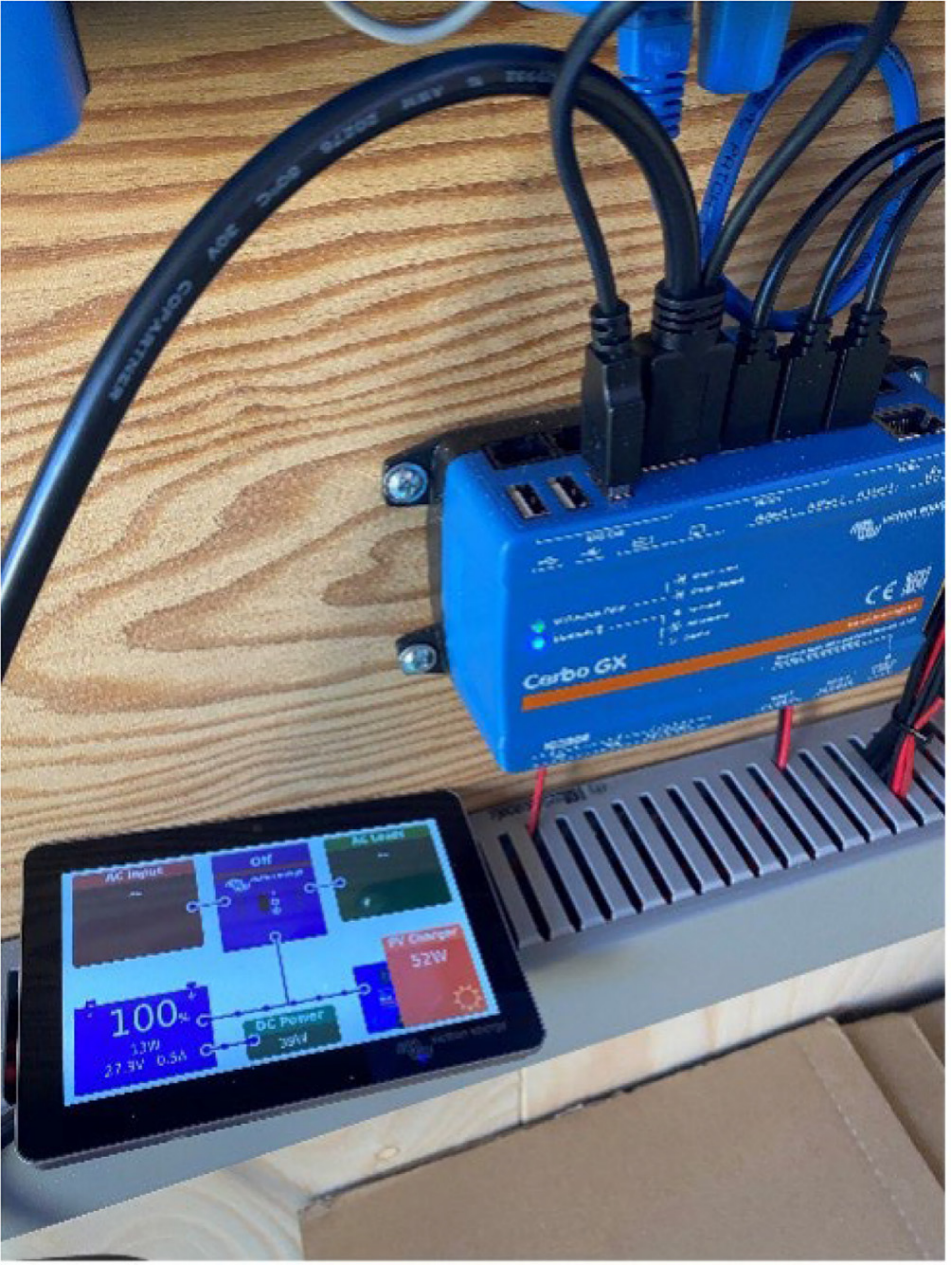
Connecting to the ARC-MO via Bluetooth.

6. Connect Display, here you will get an overview of the overall system.

7. With the VictronConnect app you will be able to communicate with all components via Bluetooth (Fig. 20).

### Operations check of the system from remote

Data collection by the various sensors (atmosphere, land, ocean) and status can be monitored remotely via the Greenland Integrated Observing System (GIOS) homepage (https://gios.org) using the Grafana dashboard (Fig. 17). We are continuing to update information on the system health e.g. battery voltage, pitch and roll for ocean ADCP mooring, data transmission etc.

### Concerning the server and communication

Check this out when visiting the field:

1. Be sure the server BIOS is configured to power on after a power breaks. Press F1 on the keyboard to set/change BIOS settings. Test it works by shutting down the server, remove power and attach power again.
2. Check an FTP site is running on the server.
3. In the Windows Scheduler the console application for data transmission must be scheduled to run once a day.
4. Turn on power for the Iridium modem, using the relay. Be sure this works, and inspect the antennae signal on the Iridium modem.
5. Open the latest transmission log file, to check data transmission.
6. If any communication problems exist, check the patch cables going to the switch from the server, data loggers and Iridium modem. If it looks fine, try to “ping” the different devices (Campbell data logger, Iridium modem etc. by IP address).
7. Make a copy of data on the server hard disk, or take a full backup of the disk
8. Backup container power system data (from internal SD Card).
9. Access the file system, or use remote control, on servers in connected hubs, to check the long-range Wi-Fi connection.

### Atmosphere

Safety instructions and maintenance of the atmospheric system when visiting in the field:

1. Inspect the cables/cable protection running to/from the mast to/from the container.
2. Perform a visual inspection of the mast, the guy wires, and all parts incl. cables at the mast.
3. Adjust the guy wires if needed.
4. When working on the mast please use safety gear (harness and hard hat)
5. Avoid people working directly below the instruments when working in the mast. If that is not possible, please make sure that all are wearing hard hats.
6. Use tools with care when working in the mast.
7. Inspect all instruments at the mast.
8. Clean and adjust the radiation sensors and other instruments at the mast if needed (Do NOT clean the ultrasonic anemometer, the windvane, and the cup anemometer).
9. Clean and adjust the gas analyzers if needed.
10. Perform a zero/span check of the gas analyzers.
11. Copy and empty Campbell CR1000 datalogger microSD card.
12. Check the internal Campbell CR1000 datalogger battery voltage and replace if needed.
13. Important: the cup anemometer must be exchanged due to calibration every 3 years.

### Land

Safety instructions and maintenance of the eddy covariance installation when visiting in the field:

1. Perform physical inspection of all tripod components including sensor mounts and cabling.
2. Make sure tripod is levelled, adjust if needed.
3. Secure and tighten tripod guywires.
4. Inspect (clean, level and adjust) radiation sensors.
5. If visiting during presence of snow cover perform manual snow depth measurement in the snow range sensor area of view.
6. Inspect local communication between eddy covariance system and local computer.
7. Download high frequency eddy covariance data and empty CR6 logger micro SD card storage.
8. Check internal CR6 logger battery voltage, if needed replace.
9. Replace inlet vortex filter.
10. Perform zero and span check of gas analyzer.
11. Backup local computer HDD.
12. Backup container power system data.
13. Download images from automatic camera overlooking eddy covariance tower, clean memory card and replace batteries.

### Time-lapse cameras

Safety instructions and maintenance of the time-lapse camera installation when visiting in the field:

1. Perform physical inspection of all camera and solar panel mounting frames and re-tighten fittings and replace any damaged cables.
2. Inspect (clean, level and adjust) cameras.
3. If visiting during presence of snow cover perform manual snow depth measurement in the camera area of view.
4. Inspect local 5 V power supply communication between camera and battery controller
5. Inspect WiFi communication between cameras and the terrestrial container.
6. Download image data from SD card in Raspberry Pi using WiFi connection.
7. Download logging data from SD card in Battery controller.
8. Check battery voltage, if needed replace.

### Lake and streams

Safety instructions and maintenance of the limnic system when visiting in the field:

1. Check for loose cables and damage to the solar panel.
2. Check that all loggers (i.e. water temperature (TinyTag; Gemini data loggers UK), light intensity (HOBO instruments, USA), specific conductivity (HOBO instruments, US), dissolved oxygen (DO; miniDOT, PME instruments, USA) and level logger transducer (Solinst, Canada)).
3. Upload data from the loggers, replace batteries where relevant, and re-position the loggers in the same positions.
4. Check for loose cables and the mounting systems from solar panels to battery, and from battery to multi-sensor sondes.
5. Upload data and replace batteries if relevant. At the end of the season, bring back the multi-sensor sonde to laboratory.
6. For cameras, follow the list from 1 to 8 above (Safety instructions and maintenance of the time-lapse camera installation when visiting in the field).

### Ocean

Safety instructions and maintenance of the ARC-MO (ocean unit) system when visiting in the field:

1. Check that all protective piping from the container to the sea are intact.
2. In case ocean sensors and/or batteries need replacement use standard ocean mooring procedures e.g., acoustic release the mooring instruments from the anchor, replace instrument with new calibrated sensors fully charged batteries on the inductive mooring line.
3. Inspect the inductive mooring line, buoyancy, and swivels for damage, wear and tear.
4. Connect a newly serviced releaser with fully charged batteries to a new anchor and lower it to the seafloor. Make sure not to damage the cable under operations.
5. Check connection to the ocean mooring in the central ARC-MO hub and ensure data is transmitted and health conditions are fine.
6. Ensure standard safety procedures are followed when operating at sea.
7. Download and backup data from instruments.

## Validation and characterization

The extreme climate, the sparse infrastructure and the costly logistics make it challenging to work in the Arctic and to collect data year-round, especially during dark winter conditions. The new ARC-MO units will be an important asset to collect measurements across the landscape at times and locations where traditional logistics are prohibitively challenging. With an aim to eventually upscale long-term data collection (e.g. https://g-e-m.dk) to entire Greenland, our ARC-MO units to function as critical ground truth validation for ongoing and future satellite upscaling. An important next step in this approach will be the placement of ARC-MO units from South Greenland (60°N) to North Greenland (81°N) along climatic gradients. As the temperature gradients over a 100 km west to east section from the ocean to the Greenland ice sheet can be as steep as the temperature difference over a 2700 km south to north section, we plan to place units from the Greenland ice sheet to the outer coast (east–west) as well. This will provide a novel study approach covering both spatial and temporal variability. The ARC-MO units will be central in the Greenland Integrating Observing System (GIOS, https://gios.org).

Capabilities (and limitations) of the ARC-MO hardware

• Capable of operating under harsh Arctic conditions.
• Capable of autonomous data collection from air, land, rivers and ocean.
• Capable of transferring near real-time data.
• Flexible system allowing for different sensor packages.
• A limiting factor for the hardware is the power production during the dark winter months where solar panels do not function and where units rely on power generation from the windmill and internal batteries.
• A limiting factor for the real-time data transfer is the cost. At present we are not transmitting large size files, e.g. photos, and all instruments’ raw data. These must be collected on the annual service visit to the units.

### Future improvements

Due to very poor sea ice conditions during deployment unusual high numbers of polar bears were observed in the area. Besides very close encounters between polar bears and scientists (luckily without severe injuries for bear and scientists) this resulted in polar bear damage on some cables and instruments. We are working on an improved ways to protect our instruments from polar bears. In the present setup, cables running on ground from the container to mast/instruments were all protected by “armored hose” or “steel enforced hose”. These have proven successful for smaller wildlife such as foxes and muskoxen. We also need to increase battery capacity in our sub-sea inductive mooring as low temperatures under Arctic conditions are reducing battery capacity more than expected for this system. New satellite systems and reduced data transmission costs and power usage (e.g. STARLINK or Swarm) open up the possibility for transmitting raw data in near real time.

## Ethics statements

Not relevant.

## Declaration of Competing Interest

The authors declare that they have no known competing financial interests or personal relationships that could have appeared to influence the work reported in this paper.

## References

[1] AMAP (2021).

[2] Kulmula M. (2018). Building a global Earth observatory. Nature.

[3] Metcalfe D.B., Hermans T.D.G., Ahlstrand J., Becker M., Berggren M., Björk R.G., Björkman M.P., Blok D., Chaudhary N., Chisholm C., Classen A.T., Hasselquist N.J., Jonsson M., Kristensen J.A., Kumordzi B.B., Lee H., Mayor J.R., Prevéy J., Pantazatou K., Rousk J., Sponseller R.A., Sundqvist M.K., Tang J., Uddling J., Wallin G., Zhang W., Ahlström A., Tenenbaum D.E., Abdi A.M. (2018). Patchy field sampling biases understanding of climate change impacts across the Arctic. Nat. Ecol. Evol..

[4] *INTERACT 2020. INTERACT Station Catalogue – 2020. Eds.: Arndal, M.F. and Topp-Jørgensen, E.DCE – Danish Centre for Environment and Energy, Aarhus University, Denmark. 190 p.*

[5] Soltwedel T., Bauerfeind E., Bergmann M., Budaeva N., Hoste E., Jaeckisch N., von Juterzenka K., Matthiessen J., Mokievsky V., Nöthig E.-M., Quéric N.-V., Sablotny B., Sauter E., Schewe I., Urban-Malinga B., Wegner J., Wlodarska-Kowalczuk M., Klages M. (2005). HAUSGARTEN: Multidisciplinary investigations at a deep-sea, long-term observatory in the Arctic Ocean. Oceanography.

[6] Trowbridge J., Weller R., Kelley D., Dever E., Plueddemann A., Barth J.A., Kawka O. (2019). The ocean observatories initiative. Front. Mar. Sci..

[7] Dmitrenko I.A., Kirillov S.A., Rudels B., Babb D.G., Myers P.G., Stedmon C.A., Bendtsen J., Ehn J.K., Pedersen L.T., Rysgaard S., Barber D.G. (2019). Variability of the pacific-derived arctic water over the southeastern Wandel Sea Shelf (Northeast Greenland) in 2015–2016. J. Geophys. Res.-Oceans.

[8] Duke P.J. (2021). Seasonal marine carbon system processes in an Arctic coastal landfast sea ice environment observed with an innovative underwater sensor platform. Elem Sci. Anth..

[9] Rysgaard S., Glud R.N. (2007). Carbon cycling in Arctic marine ecosystems: Case study - Young Sound. Medd Greenland. Bioscience.

[10] Meltofte H., Christensen T.R., Elberling B., Forchhammer M., Rasch M. (2008). High-Arctic ecosystem dynamics in a changing climate. Adv. Ecol. Res..

